# Pharmacological targeting of TNS3 with histone deacetylase inhibitor as a therapeutic strategy in esophageal squamous cell carcinoma

**DOI:** 10.18632/aging.203091

**Published:** 2021-05-28

**Authors:** Yang Shi, Zheng Xiang, Huiyu Yang, Suliman Khan, Ruizhe Li, Siran Zhou, Saif Ullah, Jiyu Zhang, Bingrong Liu

**Affiliations:** 1 Department of Gastroenterology, The First Affiliated Hospital of Zhengzhou University, Zhengzhou 450052, China; 2 State Key Laboratory of Esophageal Cancer Prevention and Treatment, Zhengzhou University, Zhengzhou 450052, China; 3 Department of Pathology, The First Affiliated Hospital of Zhengzhou University, Zhengzhou 450052, China; 4 Department of Pathology, Henan Provincial People's Hospital, Zhengzhou University People's Hospital, Henan University People's Hospital, Zhengzhou 450003, China; 5 Department of Cerebrovascular Diseases, The Second Affiliated Hospital of Zhengzhou University, Zhengzhou 450000, China

**Keywords:** histone acetylation, histone deacetylase inhibitors, LMK-235, esophageal squamous cell carcinoma, tensin-3

## Abstract

Histone acetylation which regulates about 2-10% of genes has been demonstrated to be involved in tumorigenesis of esophageal squamous cell carcinoma (ESCC). In this study, we investigated the treatment response of ESCC to selective histone deacetylase inhibitor (HDACi) LMK-235 and potential biomarker predicting the treatment sensitivity. We identified tensin-3 (TNS3) which was highly over-expressed in ESCC as one of the down-regulated genes in response to LMK-235 treatment. TNS3 was found positively correlated with the tumor malignancy and poor prognosis in the patients. Silencing *TNS3* significantly inhibited ESCC cell proliferation both *in vitro* and *in vivo*, sensitizing the treatment response to LMK-235. Our findings provide an insight into understanding the oncogenic role of TNS3 in ESCC and its clinical application for HDAC targeted therapy of ESCC.

## INTRODUCTION

Esophageal cancer (EC) is the fourth most common gastrointestinal (GI) cancer worldwide, etiologically consists of two main subtypes: esophageal squamous cell carcinoma (ESCC) and adenocarcinoma (EAC). There were 604,100 EC cases (ESCC, about 90%; EAC, about 10%) in 2020, estimated by International Agency for Research on Cancer (IARC) [1]. Despite the advancement in diagnosis and therapeutic maneuvers, the prognosis of ESCC patients are still dismal. It is therefore imperative to identify biomarkers to recognize unique biological features of ESCC so as to guide and improve individualized treatment.

Over the past several decades, it has become evident that epigenetic disturbances play a crucial role in oncogenesis [2]. Most importantly, abnormalities of histone acetylation have been reported to be involved in ESCC development and progression by modulating chromatin remodeling, which results in differential regulation of critical genes associated [3]. Histone acetylation is controlled by two families of enzymes; histone acetyltransferase (HATs) and histone deacetylases (HDACs) [4, 5]. The HDAC families consist of four classes (I, II, III, and IV), and the subgroups of II constitutes IIa (HDAC4, 5, 7, and 9) and IIb (HDAC6 and 10) [6], one of which HDAC4 has been found linked with malignant phenotypes of ESCC [7]. Previous reports have concluded that the disturbed equilibrium of histones acetylation is associated with a variety of malignancies, including ESCC, which can be used as potential targets [8–10].

Since the approval of Vorinostat for refractory cutaneous T-cell lymphoma (CTCL) by the Food and Drug Administration (FDA), numerous HDAC inhibitors (HDACis) have received serious attention during past few years [11, 12]. Given that the numerous side effects caused by commonly used pan-HDACis, selective HDACis with equipotent cytotoxicity are desirable [13, 14]. Extending further, hydroxamate-based HDACi LMK-235, specific for HDAC4 and HDAC5, has been demonstrated more promising as compared to pan-HDACi against several cancer cell lines [15]. However, drug resistance of HDACi limits the therapeutic efficacy and clinical applications [16].

In this study, we compared the transcriptional landscape of ESCC treated with LMK-235, and detected a subset of significantly up and down-regulated genes, which were subsequently examined by high-content siRNA screen based on cell proliferation. We noted that tensin-3 (*TNS3*), one of the most distinct proliferation defected genes, was associated with the oncogenic clinical traits and correlated with poor prognosis in ESCC patients. Silencing *TNS3* markedly inhibited ESCC cell proliferation both *in vitro* and *in vivo*, and enhanced the treatment sensitivity to LMK-235. Our findings shed light on development of potential biomarkers for patient stratification and therapeutic targets to improve HDACi treatment efficacy for ESCC patients.

## RESULTS

### LMK-235 impairs ESCC cells viability

**Figure 1:**
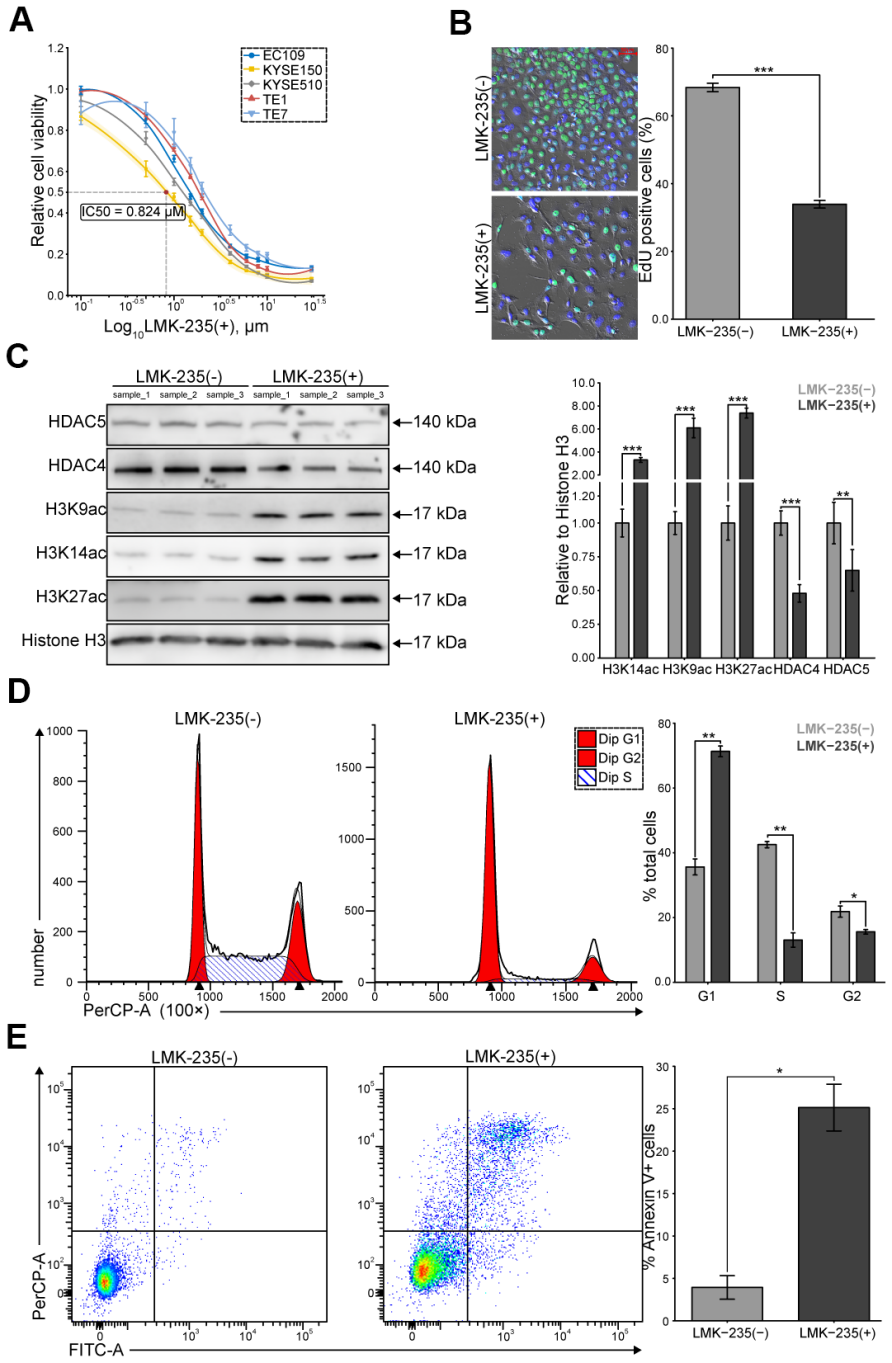
**LMK-235 inhibits ESCC cells proliferation.** (**A**) Growth curves of the five ESCC cell lines treated with LMK-235 for 48 hr. Absorbance at OD450 nm are normalized to vehicle control (0.1% DMSO; n = 5 wells/dose point). IC50 of KYSE150 is marked as the red point. (**B**) EdU incorporation assay of KYSE150 treated with LMK-235 (0.824 μM) for 48 hr. Scale bar = 50 μm. (**C**) The expressions of H3K27ac, H3K14ac, H3K9ac, HDAC4, and HDAC5 are determined by western blot in KYSE150 treated as (**B**). Histone H3 is used as the loading control. (**D**, **E**). Cell cycle distribution (**D**) and the percentage of Annexin V+ cells (**E**) of KYSE150 treated as (**B**), analyzed by flow cytometry. (**A**–**E**) Error bar denotes SEM of three replicates.

We evaluated the cytotoxicity of LMK-235 on ESCC cells, including EC109, TE-1, TE-7, KYSE150, and KYSE510. We observed dose-dependent impaired viability at 48 hr, where KYSE150 was detected as the most sensitive cells (IC50 = 0.824 μM, Figure 1A). We found that the percentage of EdU incorporation in KYSE150 was significantly reduced by LMK-235 (Figure 1B). Meanwhile, the levels of acetylated lysine residues of histone H3 (H3K27ac, H3K14ac, and H3K9ac) and class IIa HDACs (HDAC4 and HDAC5) were increased and decreased in response to the LMK-235 treatment of KYSE150, respectively (Figure 1C). Moreover, we found that G1/S transition was significantly blocked (Figure 1D), and the rates of apoptosis were increased (Figure 1E), in KYSE150 treated with LMK-235. Our data demonstrate the potential therapeutic value of LMK-235 to ESCC cells.

### LMK-235 modulates the transcriptional signatures of ESCC drivers

**Figure 2:**
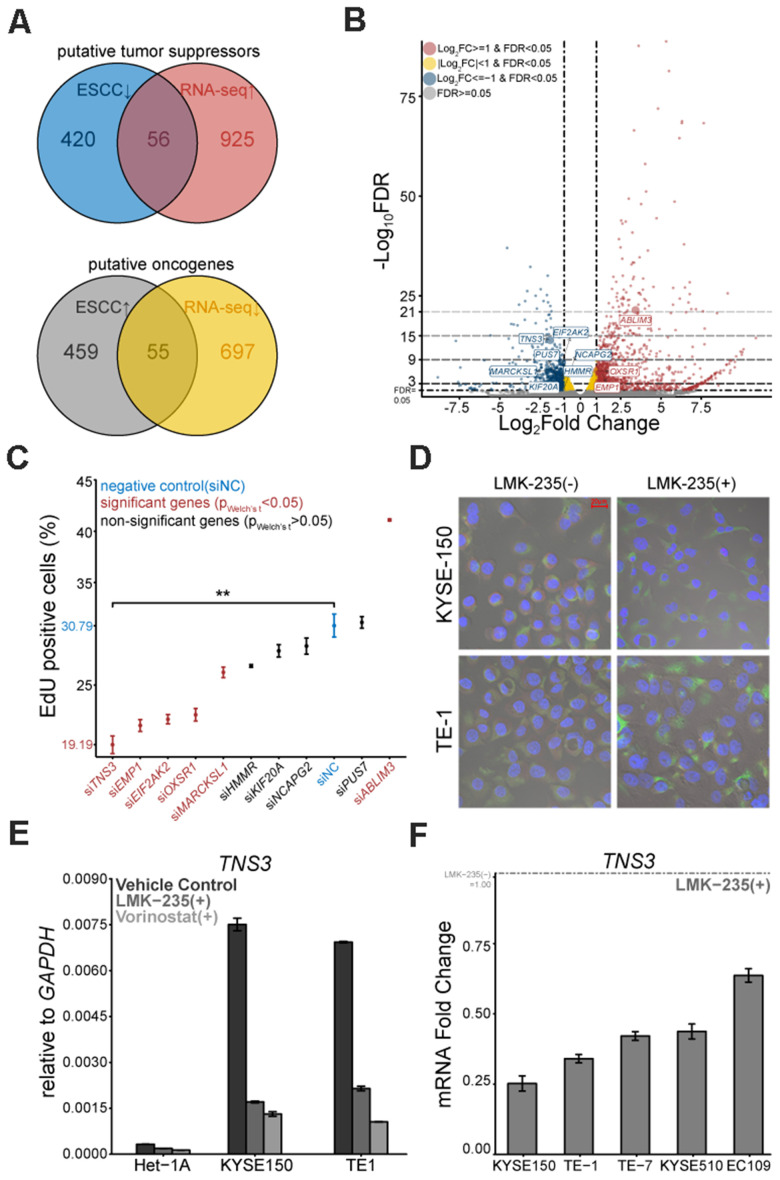
**Putative oncogene *TNS3* is mitigated by LMK-235.** (**A**) Venn diagrams of the 56 putative tumor suppressors and 55 putative oncogenes. (**B**) Volcano plot exhibits the DEGs mediated by LMK-235, including the selected 10 genes. (**C**) Loss-of-function proliferation high-content based siRNA screen of the selected 10 genes. (**D**) Immunofluorescence of TNS3 in KYSE150 and TE-1, treated with LMK-235. Red: TNS3, Green: α-Tubulin, Blue: DAPI. Scale bar = 20 μm. (**E**) qRT-PCR analysis of *TNS3* expression in Het-1A, TE-1, and KYSE150 cells treated with LMK-235 and Vorinostat. Data are normalized to *GAPDH*. (**F**) qRT-PCR analysis of *TNS3* expression in EC109, KYSE150, KYSE510, TE-1, and TE-7 cells treated with LMK-235. Data are relative to vehicle control and normalized to *GAPDH*. (**C**, **E**, **F**). Error bar denotes SEM of three replicates.

We employed RNA-seq to decipher the transcriptome of KYSE150 treated with LMK-235 (LMK-235[+]). Compared to the vehicle control (LMK-235[-]), we identified 1733 genes significantly regulated by LMK-235 (Supplementary Figure 1B and Supplementary Table 1). KEGG pathway enrichment analysis showed that a large number of these genes were involved in pathways complicated with carcinogenesis (Supplementary Figure 1C). The GSEA depicted gene signatures were impacted by LMK-235, such as nucleosome assembly and apoptosis were suppressed and activated, respectively (Supplementary Figure 1D). By integrative analysis with the differentially expressed genes (DEGs) of ESCC (183 pairs) from GEO database [17–19], we detected 56 putative tumor suppressors and 55 putative oncogenes from the 1733 genes of RNA-seq (Supplementary Figure 1E and Supplementary Table 1, Figure 2A).

### High-content based siRNA screen identifies tensin-3

Based on the intersections of genes (shown in Figure 2A) and their possible link with ESCC carcinogenesis, we selected 10 genes (Figure 2B). To determine the proliferation linked candidates, the customized siRNA library based on a high-content screen was conducted on KYSE150. EdU positive rate demonstrated that silencing *TNS3* led to the most distinct proliferation impairment (Figure 2C). We also examined these six proliferation related genes in KYSE150 and TE-1 treated with LMK-235 by qRT-PCR (Supplementary Figure 1F). Furthermore, we found that TNS3 localized in the cytoplasm of KYSE150 and TE-1, while their expressions were down-regulated by LMK-235 (Figure 2D). We detected *TNS3* expression showing comparable vulnerability to Vorinostat (SAHA) with LMK-235 (Figure 2E), and it also exhibited LMK-235-specific regulated genes across the other ESCC cells (Figure 2F).

### Bioinformatics analyses reveal the oncogenic property of *TNS3* in ESCC

**Figure 3:**
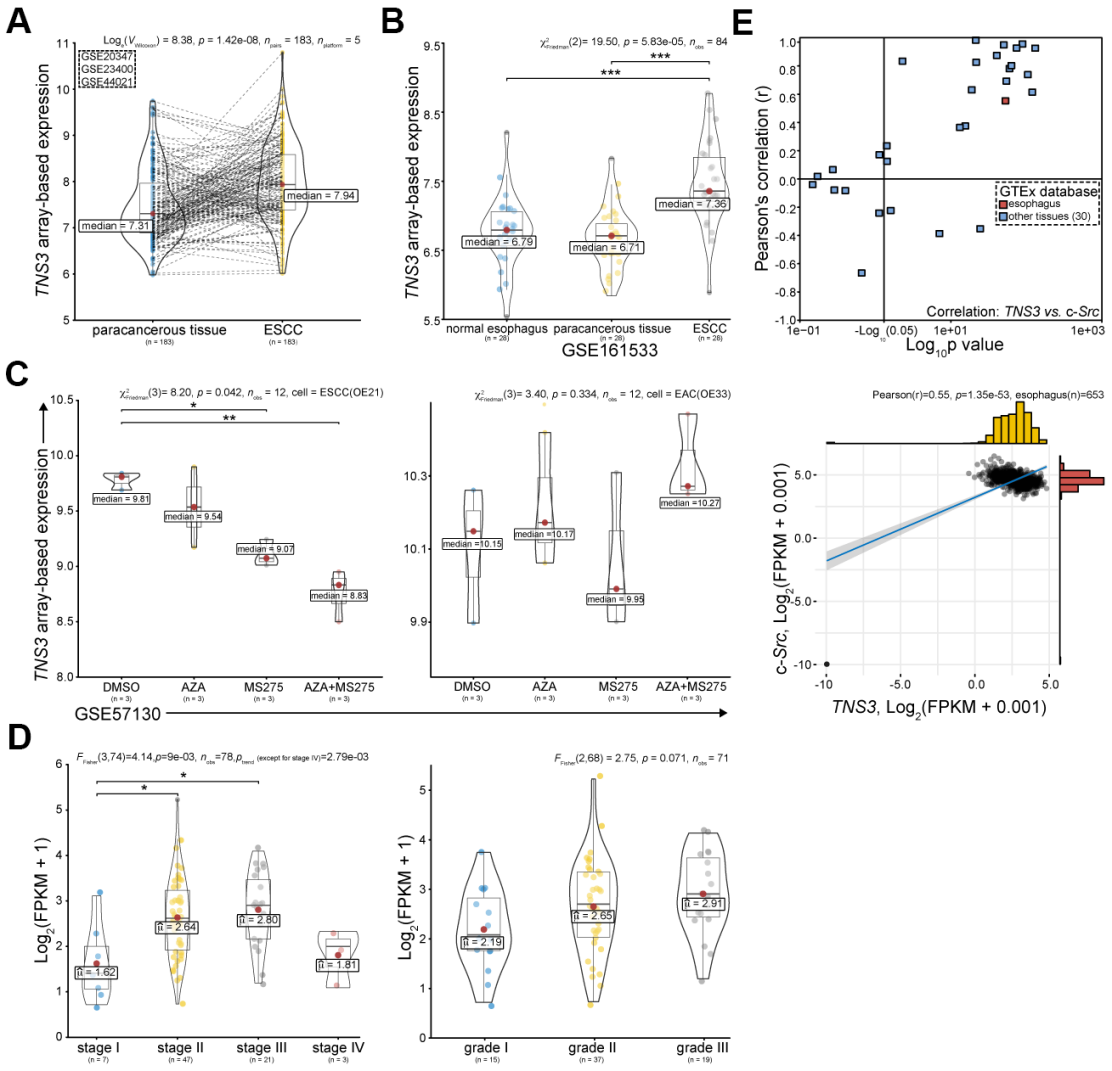
***TNS3* serves as an oncogene in ESCC based on public databases.** (**A**) *TNS3* expression in ESCC compared with the corresponding paracancerous tissues in GEO database, including GSE20347, GSE23400, and GSE44021 [17–19]. (**B**) *TNS3* expression in ESCC, the related paracancerous tissues, and related normal tissues (GSE161533). (**C**) *TNS3* expression in EC cells (ESCC OE21 and EAC OE33) treated with MS275 and AZA (GSE57130 [23]). (**D**) *TNS3* expression among different clinical TNM stage (left) and histopathological grade (right) of ESCC in TCGA database. (**E**) Correlations between *TNS3* and c-*Src* in normal tissues based on GTEx database.

The oncogenic role of *TNS3* in cancers could be mainly attributed to Src family kinases (SFKs, mainly Src), which mediate phosphorylation of SH2 domains of *TNS3* [20, 21]. However, the role of *TNS3* in ESCC has not been reported yet. We examined *TNS3* expression in ESCC using GEO and TCGA databases. We found that *TNS3* expression was significantly increased in ESCC compared to the related paracancerous tissues (Figure 3A), however, we did not find any significant difference between the normal esophagus and paracancerous tissues (Figure 3B). Nevertheless, *TNS3* expression in ESCC cell (OE21) was demonstrated as more sensitive to HDACi (MS275) than EAC cell (OE33), especially combined with Azacitidine (AZA) (Figure 3C). In addition, we detected that the higher expression of *TNS3* positively correlated with the clinical TNM stage (p trend = 2.79e-03, except for stage IV), and showed borderline significance with advanced histopathological grade (p = 0.071) (Figure 3D). We also found that positive correlations between *TNS3* and c-*Src* in most normal tissues based on GTEx database (Figure 3E).

### Over-expressed TNS3 is responsible for oncogenic phenotypes of ESCC by IHC evaluations

**Figure 4:**
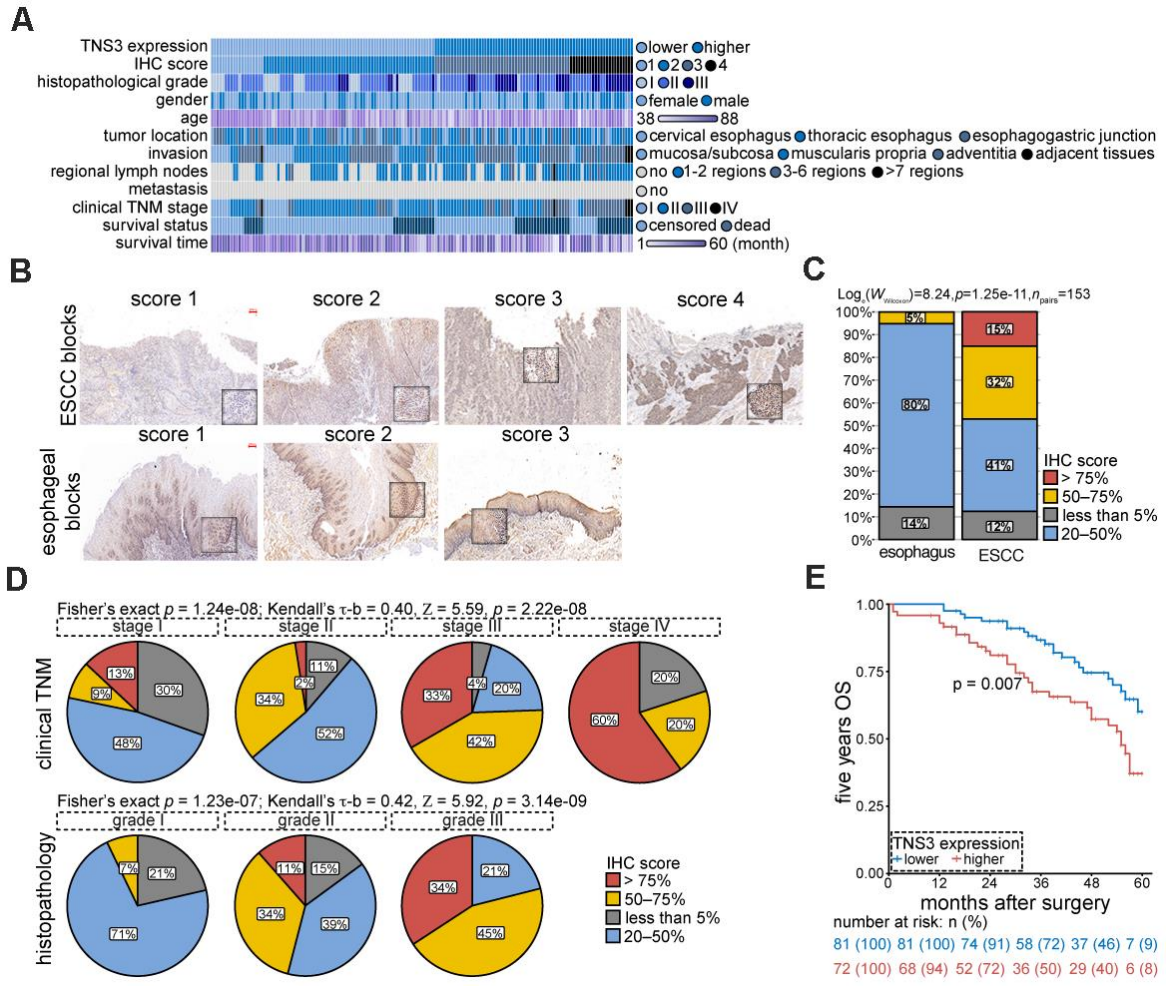
***TNS3* expression are relevant with tumorigenic properties of ESCC.** (**A**) Clinical features of the 153 ESCC patients. (**B**) Representative IHC images of TNS3 in ESCC and the normal esophageal blocks. Scale bar = 100 μm. (**C**) Quantified staining scores of (**B**) (arbitrary units). (**D**) Correlations of TNS3 immunostaining scores with clinical TNM stage (upper) and histopathological grade (bottom) of ESCC, respectively. (**E**) The five years OS rate of ESCC patients with higher and lower levels of TNS3.

We examined TNS3 expression using IHC in 153 pairs of ESCC blocks. Patients’ features were shown in Figure 4A. We observed a significant increase of TNS3 expression in tumor blocks as compared to the normal blocks (Figure 4B, 4C). The staining scores presented positive correlations with clinical TNM stage (Kendall’s τ-b = 0.40, p = 2.22e-08) and histopathological grade (Kendall’s τ-b = 0.42, p = 1.23e-07), suggesting that TNS3 may facilitate the tumorigenesis of ESCC (Figure 4D). Moreover, the five years OS rate of these patients with higher levels of TNS3 showed a dismal prognosis (Figure 4E). Collectively, these findings indicate a contribution of over-expressed TNS3 to the malignant phenotypes of ESCC.

### *TNS3* promotes proliferation of ESCC cells

**Figure 5:**
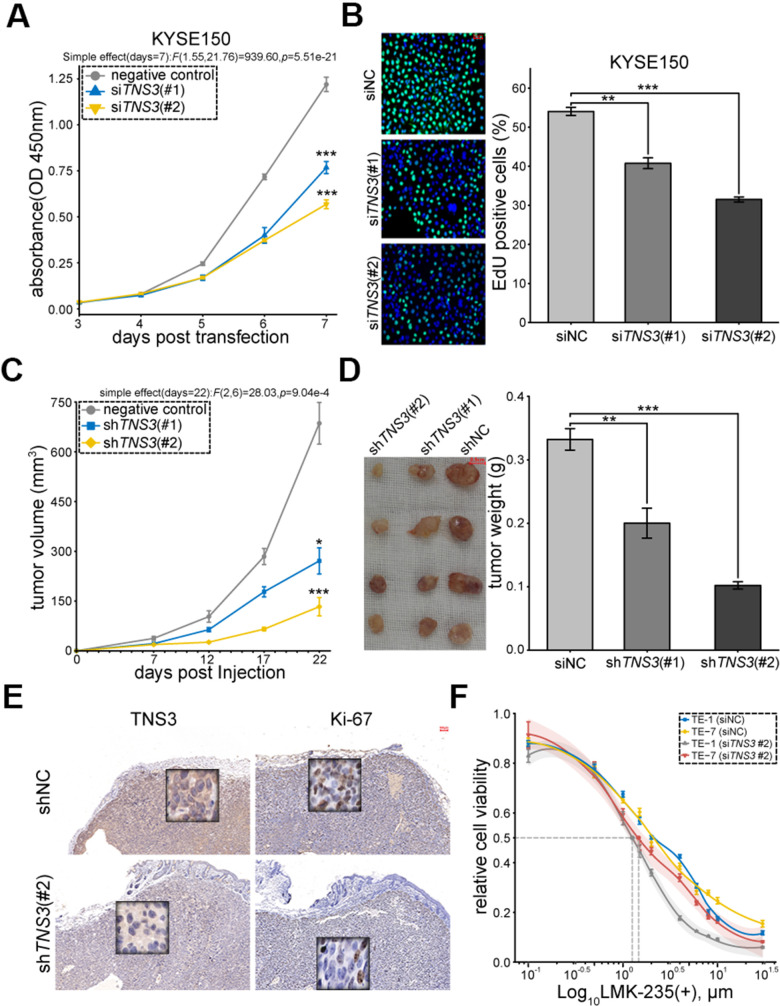
***TNS3* promotes ESCC cell proliferation.** (**A**) EdU incorporation and (**B**). CCK-8 assay of KYSE150 transfected with si*TNS3* (#1, #2) and siNC for 48 hr. Scale bar = 50 μm. (**C**) Subcutaneous tumor volumes and (**D**). weights of the xenograft mice model (expressed sh*TNS3*#1, #2, shNC). Scale bar = 0.5 cm. (**E**) Immunostaining of TNS3 and Ki-67 in the subcutaneous tumor blocks (sh*TNS3* #2 and shNC). Scale bar = 100 μm. (**F**) Growth curves of TE-1 and TE-7 treated with LMK-235 for 48 hr post-transfection with si*TNS3*(#2). Absorbance at OD450 nm are normalized to vehicle control (0.1% DMSO; n = 5 wells/dose point). IC50 of cells transfected with si*TNS3*(#2) are marked as grey (TE-1) and red (TE-7) point, respectively. (**A**–**D**, **F**) Error bar denotes SEM of three replicates.

In order to examine the biological functions of *TNS3* in ESCC, we inhibited its expression through siRNAs (Supplementary Figure 2A, 2B). The results showed impaired cell proliferation in KYSE150 and TE-1 transfected with si*TNS3* (#2) (Figure 5A, 5B and Supplementary Figure 2C, 2D). We further investigated the role of *TNS3 in vivo* using subcutaneously xenograft tumor mice model and found that KYSE150 expressing sh*TNS3* (#2) significantly reduced the tumor volume and weight compared to shNC (Figure 5C, 5D). We also detected decreased expressions of TNS3 and Ki-67 in subcutaneous tumor blocks (sh*TNS3* #2) (Figure 5E). The positive correlations of *TNS3* and *MKI67* were found in most cancers, including ESCC (Supplementary Figure 2E). Furthermore, *TNS3* knockdown could sensitize TE-1 and TE-7, which detected as higher IC50 value than KYSE150 (shown in Figure 1A), to the treatment of LMK-235 (Figure 5F).

## DISCUSSION

In spite of the improvement in conventional therapeutic strategies, the dismal five years OS rates of ESCC patients (about 15% to 20%) [1] indicate that the alternative approaches based on the underlying molecular mechanism are desirable. Since the fundamental role of HDACs are dysregulated in many cancers, epigenetic modulating agents HDACis have been tested in several tumors [22]. In order to circumvent the side effects caused by pan-HDACis, highly specific HDACis with comparable cytotoxicity are required.

In this study, we utilized the selective HDACi LMK-235, which inhibited ESCC cells proliferation, to interrogate its transcriptional effects on ESCC. By integrative analysis with ESCC in GEO database, we next identified the putative oncogenes and tumor suppressors from our RNA-seq, part of which were examined by high-content siRNA screen based on proliferation. Furthermore, we noted that the most distinct proliferation defected gene tensin-3 (*TNS3*) was upregulated in ESCC, associated with the malignant clinical characteristics, and indicated poor prognosis in patients. Subsequently *in vitro* and *in vivo* assays revealed the pro-proliferative role of *TNS3* in ESCC, inhibition of which could increase the sensitivity of tumor cells to LMK-235 treatment.

It is known that the over-expressed HDAC4 induces tumorigenesis of ESCC, while LMK-235 inhibits the growth of ESCC cells by targeting HDAC4 and 5 [7, 15]. We assessed the effect of LMK-235 in five ESCC cell lines using CCK-8 assay and demonstrated that the impaired proliferation coincides with G1-phase cell cycle arrest and increased apoptosis rate in KYSE150. Next, 10 unfeatured genes were selected and screened on the basis of KYSE150 transcriptome treated by LMK-235 and customized siRNA library about proliferation, respectively. We focused on *TNS3*, one of the most proliferation impaired gene, which was distinctly over-expressed in ESCC and down-regulated by LMK-235. We also detected that *TNS3* was more sensitive to MS275 (HDACi) in OE21 than OE33, especially when combined with AZA [23], suggesting that this gene could be a HDACi responsive gene in ESCC and a novel candidate target regulated by HDACi/AZA.

Tensins consist of four isoforms in mammals: tensin1-3 (*TNS1-3*) and cten (*TNS4*), which contain SH2 (Src Homology 2) domain and phosphotyrosine-binding (PTB) domain at their C-terminus [24, 25]. *TNS3*, identified in 2004 [26], has been reported to serve as an oncogene, which is based on Src family kinases (mainly Src) mediated tyrosine phosphorylation of its SH2 domain in several advanced cancers, such as non-small cell lung cancer, breast cancer, and melanoma [21, 27]. Although previous studies have demonstrated the tumor suppressor role of *TNS3* in untransformed MCF10A and standard HEK293 [28]; the opposing role could be due to DLC1 binding to its SH2 domain in the case of lower Src activity.

The immunostaining scores of TNS3 in ESCC blocks supported its oncogenic role, which were found: (1) significantly higher than the relevant normal sections, (2) positively correlated with histopathological grade and clinical TNM stage, (3) indicated poor prognosis. In addition, we found that *TNS3* plays a pro-proliferative role in ESCC both *in vitro* and *in vivo*, which if inhibited could enhance the sensitivity of the tumor cells to LMK-235. It is known that c-*Src* promotes cancer proliferation by activating Ras/Raf/ERK1/2 and PI3K/AKT signaling pathways [29, 30], suggesting that the oncogenic role of *TNS3* in ESCC could be involved in these pathways.

In conclusion, we found that the *TNS3* promotes ESCC cells proliferation, which could be suppressed by LMK-235. Inhibition of this gene can sensitize the treatment to LMK-235, which could serve as a potential biomarker for ESCC patients stratification and treatment targets. Further researches should focus on investigating the epigenetic landscape of ESCC treated with LMK-235 in association with down-regulated *TNS3*.

## MATERIALS AND METHODS

### Ethics statement

This study was approved by the Institutional Review Board (IRB) of the First Affiliated Hospital of Zhengzhou University (Approval #2019-KY-311). The signed informed consent were obtained from all patients. And the study was performed in accordance with the Declaration of Helsinki [31].

### Cells culture

ESCC cell lines including KYSE150, TE-1 (National Collection of Authenticated Cell Cultures, ShangHai, China), KYSE510, TE-7 (Guangzhou Jennio Biotech Co. Ltd., Guangzhou, China), and EC109 (National Infrastructure of Cell Line Resource, Beijing, China). Immortalized human esophageal squamous epithelial cell line Het-1A was purchased from ATCC (American Type Culture Collection, Manassas, VA, USA; Cat# CRL-2692^TM^). 293T cell line was purchased from (National Collection of Authenticated Cell Cultures). ESCC and 293T cell lines were cultured in RPMI-1640 and DMEM medium (HyClone™, USA), respectively, supplemented with 10% fetal bovine serum (FBS; Gibco^TM^, Thermo Scientific™, USA; Cat# 10099141C), 100 μg/ml streptomycin, and 100 units/ml penicillin (HyClone™). Het-1A cells were grown in BEGM™ Growth Medium (Lonza Walkersville Inc., USA). All cell lines were authenticated by STR and tested for mycoplasma recently.

### IC50 curves

Five ESCC cell lines, including KYSE150, KYSE510, TE-1, TE-7, EC109, were plated at 2×10^3^ cells/well into 96-well plate in five replicates, and then subjected to gradient concentrations of LMK-235 as 0.1μM, 0.5 μM, 1 μM, 1.5 μM, 2 μM, 4 μM, 6 μM, 8 μM, 10 μM, 30 μM for 48 hr. Cell viability was measured as described below. Locally weighted scatterplot smoothing (LOWESS) curve fitting [32] was performed to fit the growth curves, which enables the estimation of the half maximal inhibitory concentration (IC50) [33].

### Cell viability assays

LMK-235(Cat# HY-18998) and Vorinostat (Cat# HY-10221) were purchased from MedChemExpress (MCE, USA). KYSE150 and TE-1 cells, treated with LMK-235 for 48 hr, were plated at 2×10^3^ cells/well into 96-well plate in five replicates. Cell proliferation was measured using CCK-8 kit (Dojindo, Japan) at the following day (day 0) and every 24 hr after (up to day 4). Optical density (OD) was read at 450 nm by SpectraMax M5e Microplate Reader (Molecular Devices, CA, USA).

For EdU (5-Ethynyl-2´-deoxyuridine) incorporation assay, the above-mentioned cells were seeded into glass bottom dish (35 mm) at 5×10^4^ cells/dish in triplicate. After incubation with 10 mM EdU (RiboBio Co., Ltd, China; Cat# C10310-3) for 5 hr, nuclei were counterstained with Hoechst 33342. The percentage of proliferative cells (EdU-positive) was measured within three random fields under LSM 880 confocal microscope (Carl Zeiss, Oberkochen, Germany) using 20×objective.

### Cell cycle and apoptosis analyses

KYSE150 was treated with LMK-235 (0.824 μM) for 48 hr and then collected in cold PBS (1×10^6^ cells/ml). For cell cycle analysis, cells were fixed in 70% cold ethanol over-night and stained with 0.5 mL PI/RNase staining buffer (BD Bioscience, US; Cat# 550825) for 15 mins at room temperature (RT) before analyzing by flow cytometry (BD FACSCanto™ II). The proportions of G1, S, and G2-M phases were calculated using ModFit 5.0 (Verity Software House Inc., Topsham, ME, USA).

For apoptosis analysis, cells were resuspended in 100 μL 1×Binding Buffer, stained with 5 μL Annexin V/FITC and 5 μL PI (BD Bioscience; Cat# 556547) for 15 mins at RT, and supplemented with 400 μl 1×Binding Buffer before analyzing by flow cytometry.

### RNA extraction and real-time qRT-PCR

Total RNAs were isolated from cells using TRIzol™ reagent (Invitrogen^TM^, Thermo Scientific™; Cat# 15596026), and then the concentration and purity were examined by NanoDrop™ One^C^ Microvolume UV-Vis Spectrophotometers (Thermo Scientific™, USA). Total RNA (0.5 μg/sample) was subjected to reverse transcription (RT) using RT Master Mix with gDNA Remover (Toyobo, Japan; Cat# FSQ-301), according to the manufacturer’s instruction. Real-time quantitative polymerase chain reaction (qPCR) was performed using SYBR^®^ Green Realtime PCR Master Mix (Toyobo; Cat# QPK-201) by QuantStudio™ 5 Real-Time PCR System (Applied Biosystems, CA, USA). All primer sequences used were listed in Supplementary Table 2.

### Western blotting and antibodies

Whole-cell protein extractions (WCE) were performed using Minute Total Protein Extraction Kit (Invent Biotechnologies, USA), supplemented with protease inhibitor (Roche, Germany; Cat# 04693159001). Cell lysates were separated by sodium dodecyl sulfate-polyacrylamide gel electrophoresis (SDS-PAGE), transferred onto nitrocellulose membranes (BioTrace^TM^ NT, PALL, USA; Cat# 27376-991), blocked using Odyssey^®^ Blocking Buffer (TBS, LI-COR, Inc., USA; Cat# 927-50000) for 1 hr, and then subjected to immunoblot analyses. Total histones were extracted using Total Histone Extraction Kit (EpiQuik^TM^, EpiGentek, USA; Cat# OP-0006). Antibodies used including β-Actin (1:1000; Cell Signaling Technology, CST, USA; Cat# 4970), Histone H3 (1:1000; CST; Cat# 4499), Acetyl-Histone H3 (Lys9) (H3K9ac; 1:1000; CST; Cat# 9649), H3K14ac (1:1000; CST; Cat# 7627), H3K27ac (1:1000; CST; Cat# 8173), HDAC4 (1:1000; CST; Cat# 7628), HDAC5 (1:1000; CST; Cat# 20458), TNS3 (0.2 μg/ml; Sigma-Aldrich; Cat# HPA055338), anti-rabbit IgG (HRP-linked; 1:2000; CST; Cat# 7074).

### RNA-sequencing

Total RNAs of KYSE150, treated with LMK-235 (LMK-235[+]) and 0.1% DMSO (vehicle control, LMK-235[-]), were extracted using TRIzol™ reagent (Invitrogen^TM^). The quality of RNAs was examined by Qubit 3.0 (Thermo Scientific™, MA, USA) and Qseq100 Bio-Fragment Analyzer (BiOptic Inc., Taiwan, China). Then RNAs were used for RNA sequencing (RNA-seq) in triplicate as following: removed ribosomal RNAs (rRNAs) using QIAseq FastSelect-rRNA HMR Kit (QIAGEN, Germany; Cat# 334386), fragmented, and constructed strand-specific RNA libraries using VAHTS Stranded mRNA-seq Library Prep Kit for Illumina (Vazyme, China, Cat# NR602). All samples were sequenced by Illumina Novaseq 6000 (~ 40M, 150bp paired-end) at Epibiotek Inc. (Guangzhou, China), followed by adaptor and primer trimming.

RNA-seq data analyses were processed as the followings: reads were aligned to the GRCh38/hg38 using Hisat2 [34]. After alignment, HTSeq-Counts mapped the genome were calculated using HTSeq (version 0.11.2) [35]. The uniquely mapped reads of all samples were shown in Supplementary Figure 1A (more than 89% at least). And then the differential genes expression were calculated using DESeq2 R package [36] under the following criteria: FDR < 0.05 and |Log_2_FoldChange(FC)| > 1. FPKM (normalized fragments per kilobase per million mapped reads) was used to standardize the expression data [37]. Volcano Plot was used to present differential expressed genes, using the EnhancedVolcano R package [38]. Heatmap visualized hierarchical clustering of genes based on complete-linkage method with Euclidean distance, using pheatmap R package [39]. Gene Set Enrichment Analysis (GSEA) and Kyoto Encyclopedia of Genes and Genomes (KEGG) [40] pathway analysis were conducted using clusterProfiler R package [41]. And hallmark gene sets, such as Hallmark (h) v7.2 and Ontology (c5) v7.2, were downloaded from The Molecular Signatures Database (MSigDB) [42]. RNA-seq data have been deposited to Sequence Read Archive (SRA, PRJNA693688).

### Public databases analyses

Gene expression profiles of ESCC were downloaded from Gene Expression Omnibus (GEO) database, including GSE20347, GSE23400, GSE44021, GSE161533, and GSE57130 [17–19, 23]. The expression values of them were normalized by log_2_-transforming. Among them, five platforms [17–19] were merged to detect the DEGs by R package limma [43], including 183 ESCC and its corresponding paracancerous tissues. And the batch effects of the cross-platform were removed by R package sva [44]. Combined with genes significantly regulated by LMK-235 of our RNA-seq, we identified 56 putative tumor suppressors (down-regulated in ESCC and up-regulated by LMK-235), and 55 putative oncogenes (up-regulated in ESCC and down-regulated by LMK-235) from the DEGs. UCSC Xena platform was utilized to retrieve gene expression RNA-seq data from the following cohorts, Genotype-Tissue Expression (GTEx; primary site = 31, samples = 7858) and Genomic Data Commons (GDC) The Cancer Genome Atlas (TCGA; cancer type = 33, samples = 10966) [45].

### High-content siRNA screen and transient transfection

The high-content screen was conducted using a customized small interfering RNA (siRNA) library against the selected 10 genes in triplicate. Each mRNA was targeted by siRNA pool (50nM) consisted of three genOFF oligonucleotides (RiboBio Co., Ltd, China), of which knock-down efficiency had been validated up to 70% at least one. The viability of KYSE150 was detected by Cell-Light^TM^ EdU Apollo 488 *In Vitro* Imaging Kit (RiboBio; Cat# C10310-3) 48 hr post-transfection. Signal acquisition was taken by Acumen eX3 (TTP LabTech Ltd., Melbourn, Hertfordshire, UK): DAPI for nuclei and Apollo 488 for DNA replication in proliferating cells, which were used to calculated proliferation rates.

For transient transfection, three siRNA duplexes (#1, #2, #3) targeting human *TNS3* mRNA (GenBank accession no. NM_022748.12) and negative control (siNC) were retrieved from the siRNA library. KYSE150 and TE-1 cells were seeded at 7.5×10^4^ cells/well into 6-well plate and then transfected with siRNA at a concentration of 50 nM for 48 hr, using Lipofectamine^TM^ 3000 reagent (Invitrogen^TM^) under the manufacturer’s instruction. All sequences used were listed in Supplementary Table 3.

### Immunofluorescence

After transfected with si*TNS3* (#2) for 48 hr, KYSE150 and TE-1 were seeded onto glass coverslips, fixed in 4% paraformaldehyde (PFA; 12 min, RT), permeabilized with 0.1% Triton X-100 (10 min, RT), blocked using 2% bovine serum albumin (30 min, RT), and then incubated with primary antibodies overnight (4° C). After incubated with secondary antibodies, nuclei were counterstained with DAPI. Images were acquired by LSM 880 confocal microscope (Carl Zeiss) using 40×objective. Antibodies used including TNS3 (1.5 μg/ml; Sigma-Aldrich), α-Tubulin (1:750; CST; Cat# 3873), and Cy3- and FITC-conjugated secondary antibodies (1:500).

### Immunohistochemistry

153 pairs of formalin-fixed paraffin-embedded (FFPE) tissue blocks of treatment-naïve ESCC patients were retrieved from the Department of Pathology, the First Affiliated Hospital of Zhengzhou University from January to July 2013. The clinical features of these patients were showed in Figure 4A and Supplementary Table 5.

Immunohistochemistry (IHC) was performed using streptavidin-peroxidase based method. Staining scores were assessed by two pathologists independently according to the following criteria: (1) cytoplasmic staining of TNS3 (1:450; Sigma-Aldrich) was demonstrated positive; (2) scores were defined as percentage of positive cells × intensities: 0-25% score “1”, 25-50% score “2”, 50-75% score “3”, 75-100% score “4”. The levels of TNS3 are based on the median scores: lower level (score 1, 2) and higher level (score 3, 4). And 5 years overall survival (OS) rate of these ESCC patients was calculated based on in-patients records and follow-ups.

### Plasmids, lentivirus packaging, and stable transfection

The short hairpin RNAs target *TNS3* (sh*TNS3* #1, #2) and negative control (shNC) were cloned between Age I and EcoR I sites of pLKO.1-EGFP-puro vector (Biofeng Inc., China). For lentivirus packaging, 293T cells were seeded at 7.5×105 cells/well into 6-well plate. After 24 hr, cells were co-transfected with lentiviral expression plasmid (1 μg), psPAX2 (1.23 μg), and pMD2.G (0.74 μg) using Lipofectamine^TM^ 3000 reagent (Invitrogen^TM^). The supernatants were collected, filtered (0.45 μm PVDF; Millipore^®^, Merck), and stored at -80° C at the following two days. After transfected with lentivirus for 72 hr, KYSE150 was sorted by MoFlo XDP (Beckman, MA, USA) based on green fluorescent protein. All sequences used were listed in Supplementary Table 4.

### Mice

BALB/c Nude mice (strain code: 401, female, 4-6 week-old, 16-18g) were purchased from Charles River Laboratories (Beijing, China). Mice were randomly assigned to different cages (n = 4/cage), bred in a pathogen-free environment, and provided with free access to food and sterilized water *Ad Libitum*. Experiments were performed following principle of IRB of the First Affiliated Hospital of Zhengzhou University and the Guide for the Care and Use of Laboratory Animals, 8th edition [46].

### *In vivo* tumor growth

KYSE150 cells were transfected with sh*TNS3* (#1, #2) and shNC for 72 hr. Then the harvested cells were subcutaneously injected into the right flank of BALB/c Nude mice (n = 4/group) at 3×10^6^/100 μl/mouse. Tumor volumes were measured at indicated time, and tumor weights were measured when sacrificed. The volumes were calculated as: (Length × Width^2^)/2. Resected subcutaneous tumors were embedded in paraffin, and then sectioned at 4 μm for immunohistochemistry (IHC). Antibodies used in IHC including TNS3 (1:450; Sigma-Aldrich), and Ki-67 (1:500 CST; Cat# 9449).

### Statistical methodologies

Statistical analyses were performed using STATA software 16.0 (StataCorp, College Station, TX, USA) and RStudio (version 4.0.2). Statistical analysis was conducted using unpaired Student’s t test unless otherwise indicated. Polynomial contrast was utilized to test the trend of continuous variable within ordinal categorial variable, such as *TNS3* expression within different histopathological grade of ESCC. Two-way repeated measures Anova was employed to compare different groups transfected with si*TNS3* or sh*TNS3*, which measured more than once, including CCK-8 assay, and volumes of subcutaneous tumor. Simple effects at particular time point were calculated when there were interaction effects between time and groups. Pearson correlation coefficient (Pearson's r) was used to evaluate the correlation of gene expression, and Kendall rank correlation coefficient (Kendall’s τ-b) was used to assess the association of immunostaining scores and clinical characteristics of ESCC patients, such as histopathological grade and clinical TNM stage. Kaplan-Meier curves were utilized to compare OS rate between ‘lower’ and ‘high’ TNS3 expression (based on median score), which was calculated by log-rank test [47, 48]. FDR was computed for correcting multiple comparisons. Other statistical methods were shown in the relevant results and figures. P values were shown as: * p < 0.05, ** p < 0.01, and *** p < 0.001.

## Supplementary Material

Supplementary Figures

Supplementary Table 1

Supplementary Tables 2, 3 and 4

Supplementary Table 5
