# Supplementary material for: Pharmacological targeting of TNS3 with histone deacetylase inhibitor as a therapeutic strategy in esophageal squamous cell carcinoma
Source: Aging (Albany NY). 2021 May 28;13(11):15336–52. doi: 10.18632/aging.203091 (PMC8221360; doi:10.18632/aging.203091)
Supplement: Supplementary Figures [file aging-13-203091-s001.pdf]

SUPPLEMENTARY FIGURES

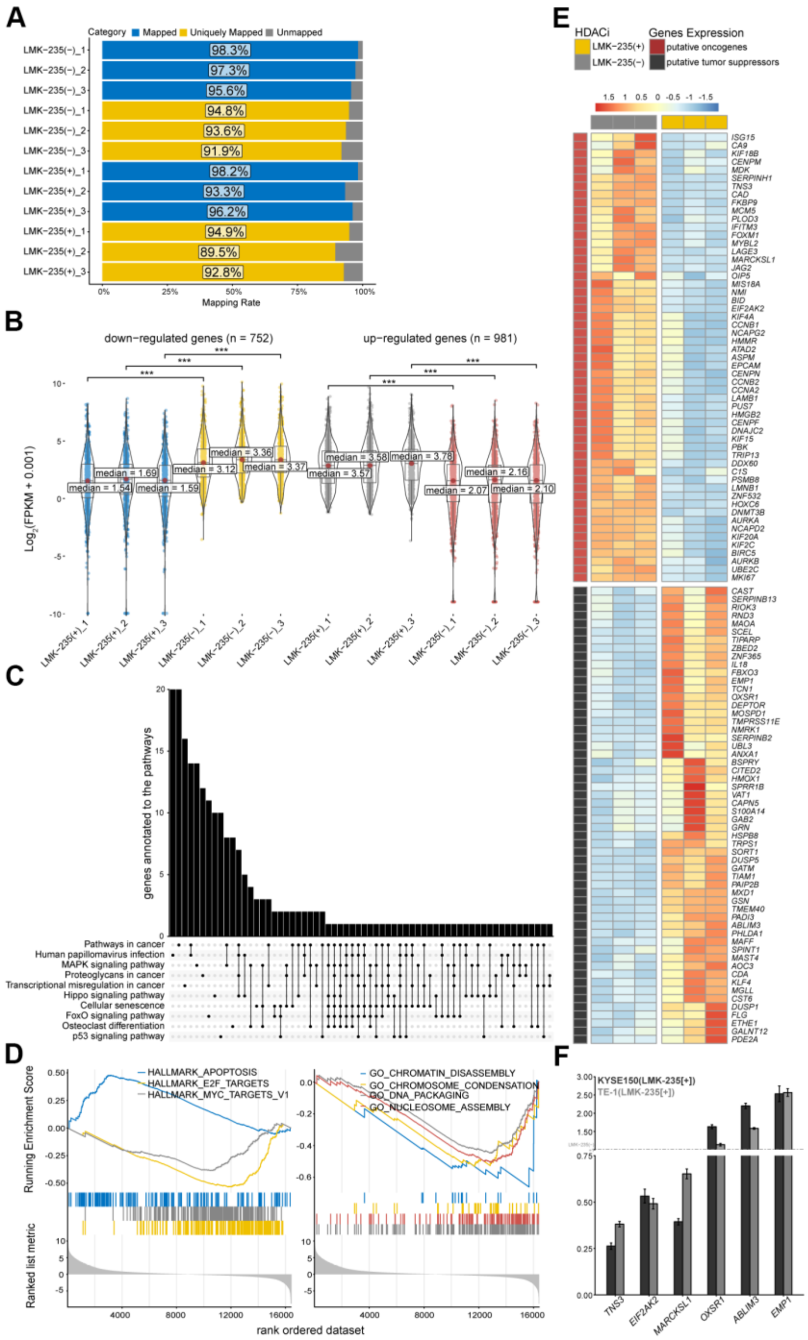

Supplementary Figure 1. The transcriptome of KYSE150 is regulated by LMK-235. (**A**) Mapping rate of RNA-seq. (**B**) Log_2(FPKM + 0.001) of genes distinctly regulated by LMK-235, including 752 down-regulated and 981 up-regulated genes. The significant criteria are set as FDR < 0.05 and $Log_2|FC| > 1$. (**C**) Top 10 KEGG pathways impacted by the treatment of LMK-235. (**D**) GSEA presents groups of genes regulated by LMK-235. The representative gene sets are displayed: Hallmark gene sets, left; Ontology gene sets, right. (**E**) Heatmaps of the genes (shown in Figure 2A), which are represented as transformed Z score of FPKM shown in each row. Labels are shown as the panels. (**F**) qRT-PCR analysis of the six growth-related genes in KYSE150 and TE-1 treated with LMK-235. Data are relative to vehicle control and normalized to *GAPDH*. Error bar denotes SEM of three replicates.

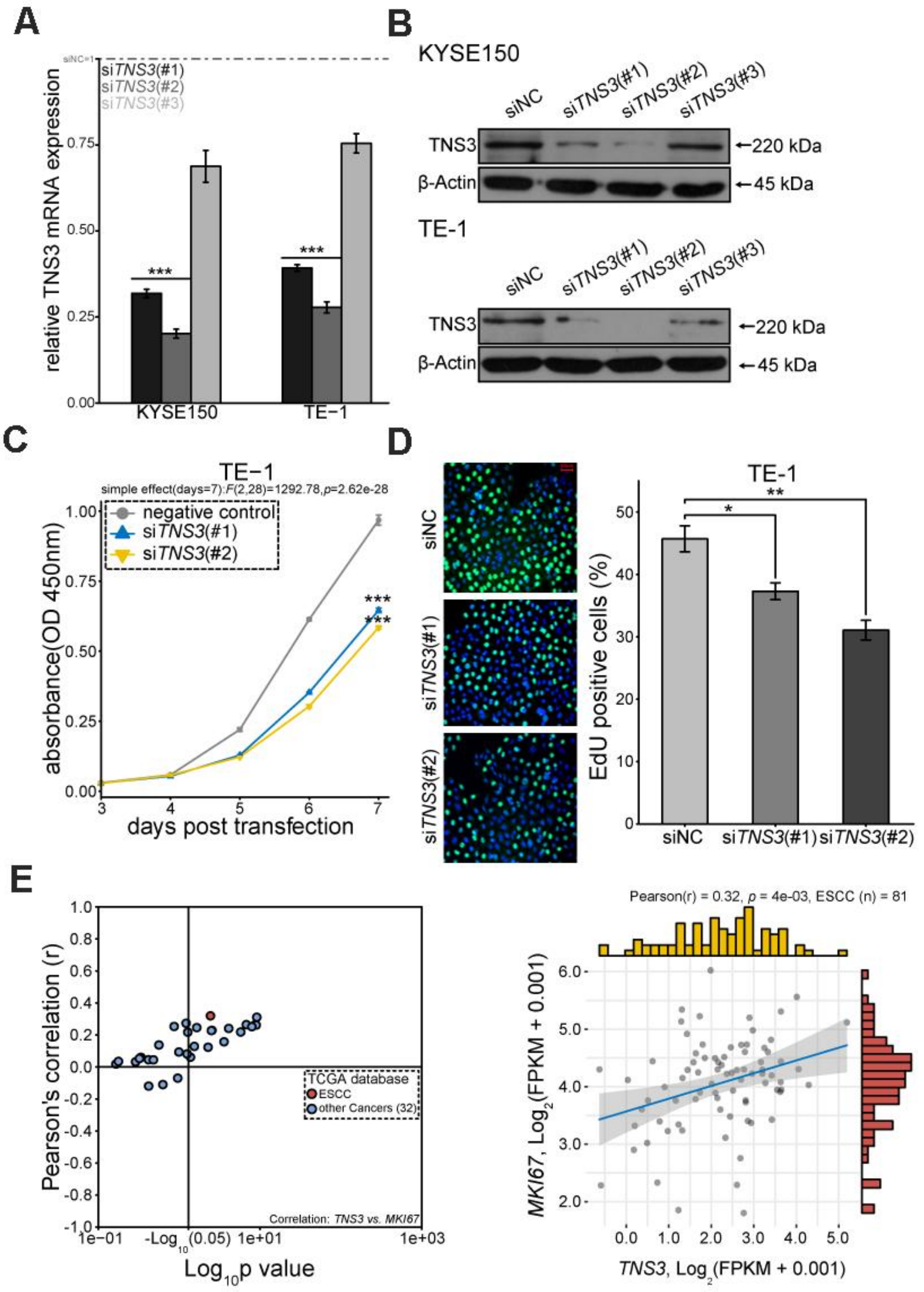

Supplementary Figure 2. *TNS3* serves as pro-proliferation factor in ESCC. (**A**) qRT-PCR analyses of *TNS3* expression in KYSE150 (left) and TE-1 (right) cells transfected with si*TNS3* (#1, #2, #3) and siNC for 48 hr. Data are relative to the negative control (siNC) and normalized to *GAPDH*. (**B**) TNS3 immunoblots of WCE from KYSE150 (upper) and TE-1 (bottom) transfected as (**A**). β-actin is used as a loading control. (**C**) Proliferation curves of TE-1 transfected with si*TNS3* (#1, #2) and siNC for 48 hr. (**D**) EdU incorporation assay of TE-1 transfected as (**C**). Scale bar = 50 μm. (**E**) Correlations between *TNS3* and *MKI67* in cancers based on TCGA database. (**A**, **C**, **D**). Error bar denotes SEM of three replicates.
