# Supplementary material for: Pharmacological targeting of TNS3 with histone deacetylase inhibitor as a therapeutic strategy in esophageal squamous cell carcinoma
Source: Aging (Albany NY). 2021 May 28;13(11):15336–52. doi: 10.18632/aging.203091 (PMC8221360; doi:10.18632/aging.203091)
Supplement: Supplementary Table 1 [file aging-13-203091-s002.doc]

**Supplementary Table 1. Genes significantly regulated by LMK-235 and genes shown in Figure 2A (putative tumor suppressors and oncogenes).**

| DEGs by LMK-235 | Putative tumor suppressors | Putative oncogenes |
| --- | --- | --- |
| STPG1 | UBL3 | SERPINH1 |
| NIPAL3 | SORT1 | UBE2C |
| HS3ST1 | EMP1 | KIF4A |
| CYP26B1 | MGLL | MARCKSL1 |
| DBNDD1 | ABLIM3 | FOXM1 |
| CAMKK1 | DUSP5 | MCM5 |
| ARHGAP33 | ZNF365 | ASPM |
| SKAP2 | VAT1 | PBK |
| LIG3 | CAST | NCAPG2 |
| ACSM3 | SCEL | CAD |
| FAM214B | MXD1 | JAG2 |
| CREBBP | GAB2 | NCAPD2 |
| WDR54 | TMPRSS11E | KIF20A |
| ITGA3 | CAPN5 | CCNB1 |
| ZFX | HSPB8 | TRIP13 |
| MAP3K14 | DUSP1 | AURKB |
| MAP3K9 | CITED2 | ATAD2 |
| KDM7A | KLF4 | MIS18A |
| ALDH3B1 | FLG | DNAJC2 |
| TTC22 | MAFF | KIF2C |
| GTF2IRD1 | TIAM1 | AURKA |
| E2F2 | FBXO3 | BID |
| JARID2 | IL18 | LMNB1 |
| CDKL5 | SERPINB13 | KIF18B |
| NCAPD2 | OXSR1 | EIF2AK2 |
| IDS | GALNT12 | PUS7 |
| HFE | MOSPD1 | CENPF |
| FYN | RIOK3 | BIRC5 |
| ANOS1 | MAST4 | MYBL2 |
| SYT7 | GSN | EPCAM |
| PLAUR | NMRK1 | FKBP9 |
| RABGAP1 | PAIP2B | ISG15 |
| ERCC1 | ETHE1 | CCNB2 |
| CALCOCO1 | PHLDA1 | CENPM |
| SLC25A39 | PDE2A | CCNA2 |
| MAMLD1 | TMEM40 | LAGE3 |
| ACPP | TRPS1 | KIF15 |
| ZMYND11 | RND3 | MKI67 |
| BID | ANXA1 | PLOD3 |
| STMN4 | HMOX1 | HMGB2 |
| WWTR1 | BSPRY | CENPN |
| CYP24A1 | GATM | HMMR |
| PRDM11 | SERPINB2 | IFITM3 |
| SNAI2 | CDA | NMI |
| ZFP64 | GRN | ZNF532 |
| SAMD4A | MAOA | LAMB1 |
| PLEKHB1 | S100A14 | PSMB8 |
| SERPINB1 | TIPARP | HOXC6 |
| OSBPL5 | DEPTOR | OIP5 |
| RNH1 | SPINT1 | MDK |
| BIRC3 | PADI3 | DNMT3B |
| VIM | ZBED2 | CA9 |
| CD44 | CST6 | DDX60 |
| AGPAT4 | AOC3 | C1S |
| IFNGR1 | TCN1 | TNS3 |
| BRD9 | SPRR1B |  |
| TBPL1 |  |  |
| GRN |  |  |
| GAB2 |  |  |
| MAP2K3 |  |  |
| SH3YL1 |  |  |
| NSMAF |  |  |
| TIMP2 |  |  |
| HOXC8 |  |  |
| RTN4R |  |  |
| TNC |  |  |
| BARX2 |  |  |
| JADE2 |  |  |
| CUL7 |  |  |
| SCML1 |  |  |
| HDAC9 |  |  |
| COL9A2 |  |  |
| KITLG |  |  |
| ADAMTS6 |  |  |
| H6PD |  |  |
| EPN3 |  |  |
| LTBP1 |  |  |
| ARID1B |  |  |
| NEDD4L |  |  |
| DKK3 |  |  |
| NFE2L3 |  |  |
| LIMA1 |  |  |
| USE1 |  |  |
| LAMA3 |  |  |
| HHAT |  |  |
| FOXC1 |  |  |
| ATP9A |  |  |
| EIF2AK2 |  |  |
| TRAF1 |  |  |
| DCBLD2 |  |  |
| MTA3 |  |  |
| USP13 |  |  |
| LAMC2 |  |  |
| CDK14 |  |  |
| RASGRF1 |  |  |
| ATP2B4 |  |  |
| UNKL |  |  |
| TARBP1 |  |  |
| MXD1 |  |  |
| SLC2A3 |  |  |
| HDAC7 |  |  |
| SLC6A16 |  |  |
| DLX3 |  |  |
| ATP2C2 |  |  |
| NGFR |  |  |
| CDON |  |  |
| CALCRL |  |  |
| ZNF76 |  |  |
| UHRF1BP1 |  |  |
| OAT |  |  |
| NTN1 |  |  |
| GLP2R |  |  |
| FAM107B |  |  |
| PPP2R5A |  |  |
| ASPM |  |  |
| FECH |  |  |
| KLF6 |  |  |
| NEO1 |  |  |
| TNFRSF1A |  |  |
| EVI5 |  |  |
| STOML1 |  |  |
| PKM |  |  |
| SYT1 |  |  |
| FGFR3 |  |  |
| LAPTM4A |  |  |
| PPP2R5B |  |  |
| MAST4 |  |  |
| NUCKS1 |  |  |
| HES2 |  |  |
| PFN2 |  |  |
| DAPP1 |  |  |
| WIPI1 |  |  |
| NDST1 |  |  |
| ASNS |  |  |
| OSBPL3 |  |  |
| TRIP13 |  |  |
| TRIB2 |  |  |
| MBD3 |  |  |
| PTPN18 |  |  |
| SREBF1 |  |  |
| SMC1A |  |  |
| HMMR |  |  |
| GSDMB |  |  |
| PTGS2 |  |  |
| PICALM |  |  |
| NOTCH3 |  |  |
| MGLL |  |  |
| ZNF532 |  |  |
| GTSE1 |  |  |
| SEMA3C |  |  |
| TTC38 |  |  |
| GRAMD4 |  |  |
| CELSR1 |  |  |
| FOSL2 |  |  |
| FRYL |  |  |
| FSCN1 |  |  |
| MOCOS |  |  |
| KIFAP3 |  |  |
| RBMS2 |  |  |
| PTPN23 |  |  |
| UNG |  |  |
| SPAG5 |  |  |
| MCAM |  |  |
| NFKB2 |  |  |
| CAPN6 |  |  |
| FBLN1 |  |  |
| MAP2 |  |  |
| EDN1 |  |  |
| TP53INP2 |  |  |
| TP73 |  |  |
| FKBP7 |  |  |
| OSBPL6 |  |  |
| SP140 |  |  |
| CDC14A |  |  |
| KEAP1 |  |  |
| RFX3 |  |  |
| COL5A3 |  |  |
| DLGAP4 |  |  |
| CXCL2 |  |  |
| ARG2 |  |  |
| STK17B |  |  |
| STRADB |  |  |
| DLG3 |  |  |
| SERTAD4 |  |  |
| GSK3B |  |  |
| GRHL2 |  |  |
| P2RX5 |  |  |
| ITGAE |  |  |
| TDRD3 |  |  |
| OXCT1 |  |  |
| CYLD |  |  |
| ZNF324 |  |  |
| FAT1 |  |  |
| SSH1 |  |  |
| APLP2 |  |  |
| NCOA1 |  |  |
| MAPRE3 |  |  |
| CAD |  |  |
| CD82 |  |  |
| BCORL1 |  |  |
| MECOM |  |  |
| WDR47 |  |  |
| PILRA |  |  |
| IGSF9 |  |  |
| AKR1B1 |  |  |
| MGST2 |  |  |
| RAD54L |  |  |
| SEPHS1 |  |  |
| TMPRSS11E |  |  |
| LPCAT2 |  |  |
| SH3BP2 |  |  |
| PTHLH |  |  |
| AURKA |  |  |
| EDEM2 |  |  |
| DNMT3B |  |  |
| EPB41L1 |  |  |
| TMEM40 |  |  |
| SMOX |  |  |
| FKBP1A |  |  |
| MAPKAPK5 |  |  |
| BIRC5 |  |  |
| OTUB2 |  |  |
| ANKRD24 |  |  |
| SLC9A1 |  |  |
| NDUFB2 |  |  |
| TFAP4 |  |  |
| EFNB1 |  |  |
| KIF4A |  |  |
| PITPNM2 |  |  |
| PUS7 |  |  |
| LAMB1 |  |  |
| ITGA6 |  |  |
| PITPNM3 |  |  |
| CCDC80 |  |  |
| PHGDH |  |  |
| UNC13D |  |  |
| TGFB2 |  |  |
| CBX5 |  |  |
| EPB41L4B |  |  |
| TBC1D2 |  |  |
| WHRN |  |  |
| TLL2 |  |  |
| SORBS1 |  |  |
| BRPF3 |  |  |
| EFHC1 |  |  |
| ZNF184 |  |  |
| SIRT1 |  |  |
| PCSK5 |  |  |
| SCD |  |  |
| NRP1 |  |  |
| HSD3B7 |  |  |
| MARCH2 |  |  |
| RANBP1 |  |  |
| SMARCB1 |  |  |
| SUSD2 |  |  |
| CYTH4 |  |  |
| LGALS1 |  |  |
| CENPM |  |  |
| KDELR3 |  |  |
| CBY1 |  |  |
| TIMP3 |  |  |
| HMOX1 |  |  |
| MCM5 |  |  |
| RASD2 |  |  |
| MYH9 |  |  |
| FOXRED2 |  |  |
| KCTD17 |  |  |
| RBX1 |  |  |
| L3MBTL2 |  |  |
| FKBP3 |  |  |
| RBM23 |  |  |
| ATP6V1D |  |  |
| PIGH |  |  |
| DHRS7 |  |  |
| TELO2 |  |  |
| DHRS2 |  |  |
| PCK2 |  |  |
| PSME2 |  |  |
| RABGGTA |  |  |
| PCIF1 |  |  |
| PROCR |  |  |
| MYBL2 |  |  |
| ADNP |  |  |
| BMP7 |  |  |
| COL20A1 |  |  |
| RNF24 |  |  |
| HM13 |  |  |
| SMAD7 |  |  |
| NOL4 |  |  |
| RIOK3 |  |  |
| ATG4A |  |  |
| MOSPD1 |  |  |
| CCDC22 |  |  |
| SMARCA1 |  |  |
| SMS |  |  |
| PHEX |  |  |
| USP11 |  |  |
| KLHL4 |  |  |
| PORCN |  |  |
| SRPX2 |  |  |
| PBDC1 |  |  |
| SLC25A15 |  |  |
| TSC22D1 |  |  |
| ZNF629 |  |  |
| GDPD3 |  |  |
| ELMO3 |  |  |
| PLLP |  |  |
| PARD6A |  |  |
| MMP15 |  |  |
| CCDC113 |  |  |
| HAS3 |  |  |
| HCFC1R1 |  |  |
| NAGPA |  |  |
| COTL1 |  |  |
| NME4 |  |  |
| HAGHL |  |  |
| ELOB |  |  |
| USP31 |  |  |
| TMC5 |  |  |
| C16orf62 |  |  |
| IQCH |  |  |
| CORO2B |  |  |
| CD276 |  |  |
| ATP8B4 |  |  |
| OIP5 |  |  |
| NIPAL2 |  |  |
| PLAT |  |  |
| NDRG1 |  |  |
| TRPS1 |  |  |
| GSDMD |  |  |
| SLC39A14 |  |  |
| TUSC3 |  |  |
| PLEKHJ1 |  |  |
| TIMM44 |  |  |
| IL27RA |  |  |
| ILVBL |  |  |
| MYH14 |  |  |
| RABAC1 |  |  |
| CARD8 |  |  |
| LIG1 |  |  |
| PLA2G4C |  |  |
| BCAT2 |  |  |
| DNASE2 |  |  |
| USF2 |  |  |
| LSR |  |  |
| SIPA1L3 |  |  |
| ETHE1 |  |  |
| CDK6 |  |  |
| DNAJC2 |  |  |
| TWISTNB |  |  |
| DNAJB6 |  |  |
| HOXA5 |  |  |
| HOXA6 |  |  |
| GRB10 |  |  |
| STX1A |  |  |
| NOD1 |  |  |
| AGFG2 |  |  |
| SERPINE1 |  |  |
| AP1S1 |  |  |
| C1GALT1 |  |  |
| PLOD3 |  |  |
| TSPAN13 |  |  |
| AHR |  |  |
| PRKAG2 |  |  |
| CLIP2 |  |  |
| NMRK1 |  |  |
| TGFBR1 |  |  |
| C5 |  |  |
| CA9 |  |  |
| GLIS3 |  |  |
| RAPGEF1 |  |  |
| ABCA2 |  |  |
| RASSF4 |  |  |
| DNMBP |  |  |
| TRDMT1 |  |  |
| VSIR |  |  |
| ACTA2 |  |  |
| LIPA |  |  |
| SFXN3 |  |  |
| KAZALD1 |  |  |
| FBXW4 |  |  |
| DKK1 |  |  |
| DNAJC12 |  |  |
| RUNDC3A |  |  |
| RGS9 |  |  |
| RNF43 |  |  |
| WNT3 |  |  |
| INTS2 |  |  |
| LGALS3BP |  |  |
| KAT2A |  |  |
| VAT1 |  |  |
| HDAC5 |  |  |
| SMURF2 |  |  |
| MMD |  |  |
| MAP2K6 |  |  |
| TNFAIP1 |  |  |
| IFT20 |  |  |
| SLAIN2 |  |  |
| CHIC2 |  |  |
| AREG |  |  |
| TBC1D9 |  |  |
| INPP4B |  |  |
| ANXA10 |  |  |
| GALNT7 |  |  |
| VWA5A |  |  |
| DTX4 |  |  |
| ATG2A |  |  |
| KMT5B |  |  |
| ARHGEF17 |  |  |
| CEP126 |  |  |
| GALNT18 |  |  |
| FBXO3 |  |  |
| ACCS |  |  |
| MDK |  |  |
| ELMOD1 |  |  |
| CORO1C |  |  |
| GLI1 |  |  |
| ELK3 |  |  |
| WNT5B |  |  |
| FOXM1 |  |  |
| MYL2 |  |  |
| CDK2AP1 |  |  |
| ARHGDIB |  |  |
| TIMELESS |  |  |
| CDCA3 |  |  |
| NT5DC3 |  |  |
| SLCO1B3 |  |  |
| BTN3A3 |  |  |
| NEDD9 |  |  |
| ADTRP |  |  |
| MCM9 |  |  |
| MAN1A1 |  |  |
| HDDC2 |  |  |
| UST |  |  |
| BACH2 |  |  |
| CAP2 |  |  |
| WASF1 |  |  |
| ALDH5A1 |  |  |
| PERP |  |  |
| ARFGEF3 |  |  |
| BICRAL |  |  |
| SENP6 |  |  |
| FAM46A |  |  |
| BRD8 |  |  |
| KIF20A |  |  |
| NNT |  |  |
| HBEGF |  |  |
| APBB3 |  |  |
| ARSB |  |  |
| CCNG1 |  |  |
| LMNB1 |  |  |
| IRX4 |  |  |
| FGF1 |  |  |
| TXNDC15 |  |  |
| ERGIC1 |  |  |
| ATP6V0E1 |  |  |
| STC2 |  |  |
| CPEB4 |  |  |
| UNC5A |  |  |
| SELENOK |  |  |
| ARL6 |  |  |
| HYAL1 |  |  |
| CBLB |  |  |
| C3orf52 |  |  |
| ATP6V1A |  |  |
| ABTB1 |  |  |
| CISH |  |  |
| SSR3 |  |  |
| SLC4A3 |  |  |
| EEF1B2 |  |  |
| KANSL3 |  |  |
| TTL |  |  |
| IL1A |  |  |
| NCL |  |  |
| CENPA |  |  |
| ST3GAL5 |  |  |
| PDCL3 |  |  |
| IL18R1 |  |  |
| FHL2 |  |  |
| PASK |  |  |
| HPCAL1 |  |  |
| SLC1A4 |  |  |
| RND3 |  |  |
| EPAS1 |  |  |
| SPR |  |  |
| PARD3B |  |  |
| MARK1 |  |  |
| QSOX1 |  |  |
| ERRFI1 |  |  |
| KCNC4 |  |  |
| DLGAP3 |  |  |
| MEF2D |  |  |
| FBXO6 |  |  |
| SLC35D1 |  |  |
| PLA2G4A |  |  |
| GADD45A |  |  |
| AGMAT |  |  |
| MAP7D1 |  |  |
| NID1 |  |  |
| AKT3 |  |  |
| KDM5B |  |  |
| UAP1 |  |  |
| KIF17 |  |  |
| P3H1 |  |  |
| PRDX1 |  |  |
| TSPAN1 |  |  |
| VAMP4 |  |  |
| IRF6 |  |  |
| CENPF |  |  |
| KPTN |  |  |
| CASC1 |  |  |
| TNFAIP3 |  |  |
| RAB32 |  |  |
| MYB |  |  |
| SGK1 |  |  |
| CTGF |  |  |
| MYL12B |  |  |
| HS1BP3 |  |  |
| ELL2 |  |  |
| CYP20A1 |  |  |
| PIGZ |  |  |
| PTPA |  |  |
| PHF19 |  |  |
| NEK6 |  |  |
| BSPRY |  |  |
| NR4A3 |  |  |
| GALNT12 |  |  |
| PGF |  |  |
| LTBP2 |  |  |
| PPP4R4 |  |  |
| KLHL29 |  |  |
| DNMT3A |  |  |
| YPEL5 |  |  |
| BCL11A |  |  |
| EPCAM |  |  |
| SLC17A5 |  |  |
| SLF2 |  |  |
| IFIT2 |  |  |
| PYROXD2 |  |  |
| MXI1 |  |  |
| CFAP58 |  |  |
| DUSP1 |  |  |
| CD274 |  |  |
| MTHFD1L |  |  |
| PLEKHG1 |  |  |
| CYSTM1 |  |  |
| ARAP3 |  |  |
| TNFSF18 |  |  |
| PLXDC2 |  |  |
| TGFBI |  |  |
| KDM3B |  |  |
| UTP20 |  |  |
| TMPO |  |  |
| DUSP4 |  |  |
| CLU |  |  |
| TNFRSF10B |  |  |
| SORBS3 |  |  |
| RDH10 |  |  |
| NCAPH |  |  |
| MND1 |  |  |
| ADCY7 |  |  |
| ECHDC2 |  |  |
| GJA3 |  |  |
| ADGRB2 |  |  |
| CLCC1 |  |  |
| ACVR2A |  |  |
| UBL3 |  |  |
| ZMIZ2 |  |  |
| FAM126A |  |  |
| INHBA |  |  |
| FKBP9 |  |  |
| ARL4A |  |  |
| DNAI1 |  |  |
| CALD1 |  |  |
| PLAU |  |  |
| CIT |  |  |
| DDX54 |  |  |
| RASSF8 |  |  |
| BHLHE41 |  |  |
| OPTN |  |  |
| IKZF4 |  |  |
| RAB9A |  |  |
| NMI |  |  |
| SLC36A1 |  |  |
| B9D2 |  |  |
| INHA |  |  |
| OBSL1 |  |  |
| FAM210B |  |  |
| WFDC3 |  |  |
| PREX1 |  |  |
| SDC4 |  |  |
| PARD6B |  |  |
| ZNFX1 |  |  |
| PMEPA1 |  |  |
| PAIP2B |  |  |
| ATP8A1 |  |  |
| BTN2A2 |  |  |
| HIST1H2BJ |  |  |
| CDKN1A |  |  |
| SOX4 |  |  |
| MT2A |  |  |
| RAP2A |  |  |
| EFNB2 |  |  |
| TMEM255A |  |  |
| GRK4 |  |  |
| SOX9 |  |  |
| MRPS7 |  |  |
| IL1B |  |  |
| INSIG2 |  |  |
| TNFSF9 |  |  |
| GPR108 |  |  |
| BMP2 |  |  |
| ID1 |  |  |
| GRPR |  |  |
| FRMD8 |  |  |
| PRDX5 |  |  |
| RRAS |  |  |
| HSPA2 |  |  |
| FAM78A |  |  |
| ZC4H2 |  |  |
| FGD3 |  |  |
| BCL11B |  |  |
| BEST3 |  |  |
| GNAI1 |  |  |
| CASD1 |  |  |
| ASPHD2 |  |  |
| APOL2 |  |  |
| RAC2 |  |  |
| APOBEC3F |  |  |
| KRT17 |  |  |
| CPA4 |  |  |
| DOCK4 |  |  |
| CDHR3 |  |  |
| VGF |  |  |
| DNAJB9 |  |  |
| GAD1 |  |  |
| TMOD2 |  |  |
| CHAC1 |  |  |
| PALLD |  |  |
| SPCS3 |  |  |
| E2F8 |  |  |
| DCTD |  |  |
| PIMREG |  |  |
| SAT1 |  |  |
| RSPH3 |  |  |
| ZSWIM6 |  |  |
| GDF15 |  |  |
| PGPEP1 |  |  |
| JUND |  |  |
| TNNI2 |  |  |
| ATXN10 |  |  |
| RBBP8NL |  |  |
| ASS1 |  |  |
| POMT1 |  |  |
| ARHGEF16 |  |  |
| HIP1R |  |  |
| ZNF236 |  |  |
| AKAP12 |  |  |
| EPS8L1 |  |  |
| F12 |  |  |
| GFPT2 |  |  |
| AOC3 |  |  |
| AOC2 |  |  |
| TNS4 |  |  |
| PRKAB2 |  |  |
| RFX1 |  |  |
| SLC6A11 |  |  |
| NUP210 |  |  |
| FCRLA |  |  |
| ENOSF1 |  |  |
| PER2 |  |  |
| LANCL2 |  |  |
| ITGB4 |  |  |
| UNK |  |  |
| DLG4 |  |  |
| PCBD2 |  |  |
| FLOT2 |  |  |
| ALDH3B2 |  |  |
| TESMIN |  |  |
| PPP1R3D |  |  |
| ZBED3 |  |  |
| TMCC2 |  |  |
| RNF128 |  |  |
| BTBD2 |  |  |
| C1QTNF6 |  |  |
| FAM83F |  |  |
| BTG1 |  |  |
| DYDC2 |  |  |
| AMPD3 |  |  |
| MICALCL |  |  |
| MICAL2 |  |  |
| RRAS2 |  |  |
| RNF122 |  |  |
| LOXL2 |  |  |
| CTIF |  |  |
| CCNB1 |  |  |
| IRAK2 |  |  |
| MEIS2 |  |  |
| DPH6 |  |  |
| VAV3 |  |  |
| PTPN22 |  |  |
| SORT1 |  |  |
| VTCN1 |  |  |
| FST |  |  |
| IL15RA |  |  |
| CABLES1 |  |  |
| EMP1 |  |  |
| CDCA8 |  |  |
| AGO4 |  |  |
| TCN1 |  |  |
| GGACT |  |  |
| DZIP1 |  |  |
| ETS1 |  |  |
| SLC37A2 |  |  |
| NREP |  |  |
| ANXA1 |  |  |
| AGTPBP1 |  |  |
| PSAT1 |  |  |
| ADAM19 |  |  |
| TES |  |  |
| TROAP |  |  |
| PAN2 |  |  |
| ESPL1 |  |  |
| ZC3H10 |  |  |
| STX11 |  |  |
| PRADC1 |  |  |
| RAB11FIP5 |  |  |
| KIAA0513 |  |  |
| CCDC102A |  |  |
| ZNF670-ZNF695 |  |  |
| PCNX2 |  |  |
| ABCB10 |  |  |
| NPL |  |  |
| LAMC1 |  |  |
| SP110 |  |  |
| SERPINE2 |  |  |
| WNT10A |  |  |
| TMBIM1 |  |  |
| FLNB |  |  |
| TBC1D4 |  |  |
| THSD1 |  |  |
| PHF11 |  |  |
| SCEL |  |  |
| SPRY2 |  |  |
| TNS3 |  |  |
| IL6 |  |  |
| ZDHHC4 |  |  |
| MTHFS |  |  |
| TACO1 |  |  |
| BIN1 |  |  |
| KIAA0368 |  |  |
| KLF4 |  |  |
| TOR1A |  |  |
| FAM129B |  |  |
| STXBP1 |  |  |
| XPA |  |  |
| ANP32B |  |  |
| LMX1B |  |  |
| MYC |  |  |
| ARHGEF39 |  |  |
| TMEM63B |  |  |
| KIAA0319 |  |  |
| TUBB2A |  |  |
| UQCC2 |  |  |
| IER3 |  |  |
| ATAT1 |  |  |
| TPMT |  |  |
| FGFBP1 |  |  |
| SYTL2 |  |  |
| DDX60 |  |  |
| TMPRSS4 |  |  |
| BTG4 |  |  |
| MMP13 |  |  |
| TMPRSS13 |  |  |
| CASP1 |  |  |
| SQOR |  |  |
| LRRC49 |  |  |
| TUBGCP4 |  |  |
| ITPKA |  |  |
| SMAD6 |  |  |
| ZNF280D |  |  |
| ARHGAP29 |  |  |
| SLC44A5 |  |  |
| CYP1B1 |  |  |
| SLC5A6 |  |  |
| LOXL4 |  |  |
| DUSP5 |  |  |
| ZNF365 |  |  |
| TET1 |  |  |
| DNA2 |  |  |
| AOX1 |  |  |
| CARF |  |  |
| CDK15 |  |  |
| PARP9 |  |  |
| GLCE |  |  |
| PARP16 |  |  |
| PCDH10 |  |  |
| PRDM5 |  |  |
| BMP2K |  |  |
| PPP3CA |  |  |
| RGS3 |  |  |
| ERP27 |  |  |
| GABARAPL1 |  |  |
| ETFBKMT |  |  |
| C1RL |  |  |
| CLSTN3 |  |  |
| AMIGO2 |  |  |
| GLIPR1 |  |  |
| PHLDA1 |  |  |
| DUSP6 |  |  |
| LUM |  |  |
| AMDHD1 |  |  |
| SDSL |  |  |
| MMAB |  |  |
| TCHP |  |  |
| ZIC5 |  |  |
| CDH24 |  |  |
| FRMD6 |  |  |
| JDP2 |  |  |
| DISP2 |  |  |
| NCOA2 |  |  |
| TPM1 |  |  |
| IGF1R |  |  |
| ARRDC4 |  |  |
| PIF1 |  |  |
| ULK3 |  |  |
| PCSK6 |  |  |
| HAPLN3 |  |  |
| ABHD2 |  |  |
| MFGE8 |  |  |
| ZNF710 |  |  |
| SH3GL3 |  |  |
| NKD1 |  |  |
| NLRC5 |  |  |
| MAP1LC3B |  |  |
| TLDC1 |  |  |
| TCF25 |  |  |
| TAF4B |  |  |
| GAREM1 |  |  |
| TP53 |  |  |
| TRIM65 |  |  |
| PMAIP1 |  |  |
| ERBB2 |  |  |
| SAMD1 |  |  |
| TPGS1 |  |  |
| PLPP2 |  |  |
| PFKL |  |  |
| DUS3L |  |  |
| TMEM91 |  |  |
| IFITM3 |  |  |
| PGGHG |  |  |
| EVI5L |  |  |
| PADI3 |  |  |
| ARHGEF19 |  |  |
| C1orf216 |  |  |
| PLK4 |  |  |
| MAP3K6 |  |  |
| SYTL1 |  |  |
| BCL10 |  |  |
| CYR61 |  |  |
| TINAGL1 |  |  |
| KIF2C |  |  |
| SLC44A3 |  |  |
| ZNF697 |  |  |
| CTTNBP2NL |  |  |
| NME7 |  |  |
| MGST3 |  |  |
| UFC1 |  |  |
| NUF2 |  |  |
| HDGF |  |  |
| XPR1 |  |  |
| RGL1 |  |  |
| TUFT1 |  |  |
| MINDY1 |  |  |
| ANXA9 |  |  |
| MRPL9 |  |  |
| GABPB2 |  |  |
| DYRK3 |  |  |
| HHIPL2 |  |  |
| SLC27A3 |  |  |
| FLG |  |  |
| LYST |  |  |
| ITPKB |  |  |
| WNT9A |  |  |
| PLEKHA6 |  |  |
| RHOB |  |  |
| ZNF514 |  |  |
| EXOC6B |  |  |
| SLC20A1 |  |  |
| CDCA7 |  |  |
| PHOSPHO2 |  |  |
| ABCA12 |  |  |
| ANKMY1 |  |  |
| MARCH4 |  |  |
| NXPE3 |  |  |
| PHLDB2 |  |  |
| NCEH1 |  |  |
| TCTA |  |  |
| ATP10D |  |  |
| OCIAD2 |  |  |
| PLAC8 |  |  |
| SNCA |  |  |
| CCNA2 |  |  |
| USP53 |  |  |
| CYP4V2 |  |  |
| FAM105A |  |  |
| PLK2 |  |  |
| SSBP2 |  |  |
| IQGAP2 |  |  |
| PAM |  |  |
| COMMD10 |  |  |
| ARHGAP26 |  |  |
| TNIP1 |  |  |
| TENM2 |  |  |
| PPP1R18 |  |  |
| FAXC |  |  |
| PM20D2 |  |  |
| SLC2A12 |  |  |
| SDK1 |  |  |
| CREB5 |  |  |
| NIPSNAP2 |  |  |
| MEPCE |  |  |
| NCAPG2 |  |  |
| SH3KBP1 |  |  |
| CCNB3 |  |  |
| CHST7 |  |  |
| SLC25A37 |  |  |
| PLPP5 |  |  |
| ADHFE1 |  |  |
| LRP12 |  |  |
| UGCG |  |  |
| STOM |  |  |
| GSN |  |  |
| ASTN2 |  |  |
| WDR31 |  |  |
| INPP5E |  |  |
| NOTCH1 |  |  |
| RSU1 |  |  |
| ANKRD1 |  |  |
| HTR7 |  |  |
| MKI67 |  |  |
| LRRC27 |  |  |
| ITPRIP |  |  |
| ADM |  |  |
| APIP |  |  |
| SESN3 |  |  |
| ENDOD1 |  |  |
| SERPINH1 |  |  |
| CAPN5 |  |  |
| KAT14 |  |  |
| FEZ1 |  |  |
| COMMD7 |  |  |
| KIAA1755 |  |  |
| HMGA2 |  |  |
| ITGB1 |  |  |
| TIRAP |  |  |
| VEGFC |  |  |
| FSIP1 |  |  |
| PRSS23 |  |  |
| PPP1R1C |  |  |
| IL18 |  |  |
| PIP4K2A |  |  |
| FOXO1 |  |  |
| ITPR1 |  |  |
| TMEM86A |  |  |
| BTBD11 |  |  |
| SLC2A13 |  |  |
| MAGI1 |  |  |
| AKAP6 |  |  |
| TMEM18 |  |  |
| ZNF827 |  |  |
| BICD1 |  |  |
| FBXO4 |  |  |
| DST |  |  |
| AP1S3 |  |  |
| PTPN14 |  |  |
| MGAT5 |  |  |
| GPATCH11 |  |  |
| HSPB8 |  |  |
| SETBP1 |  |  |
| TRIM36 |  |  |
| GJA1 |  |  |
| ANKRD22 |  |  |
| SLC16A12 |  |  |
| MR1 |  |  |
| CAST |  |  |
| MERTK |  |  |
| PTPRR |  |  |
| NR4A2 |  |  |
| CCDC148 |  |  |
| SLC25A27 |  |  |
| FBXL2 |  |  |
| PTPRD |  |  |
| LURAP1L |  |  |
| KCTD15 |  |  |
| LGI4 |  |  |
| GDPD1 |  |  |
| ANKRD29 |  |  |
| SDHAF4 |  |  |
| NRGN |  |  |
| ABI3BP |  |  |
| LRRK1 |  |  |
| NEIL2 |  |  |
| OBSCN |  |  |
| CXADR |  |  |
| ADAMTS1 |  |  |
| WNT7A |  |  |
| DPH3 |  |  |
| CXXC1 |  |  |
| FBXL18 |  |  |
| AK9 |  |  |
| KLF10 |  |  |
| ATP6V1C1 |  |  |
| GTF3C6 |  |  |
| PI4K2A |  |  |
| MARVELD1 |  |  |
| MOV10 |  |  |
| RHOC |  |  |
| PPM1J |  |  |
| DEPTOR |  |  |
| PSD3 |  |  |
| TIAM1 |  |  |
| SFR1 |  |  |
| PPP2R2B |  |  |
| HKDC1 |  |  |
| HK1 |  |  |
| KAT6B |  |  |
| ATAD2 |  |  |
| FBXO32 |  |  |
| DHRS4 |  |  |
| CCNB2 |  |  |
| MYO1E |  |  |
| TSC22D3 |  |  |
| ZNF618 |  |  |
| TMEM268 |  |  |
| FMNL2 |  |  |
| GAREM2 |  |  |
| BABAM2 |  |  |
| WDR66 |  |  |
| TPRG1L |  |  |
| XDH |  |  |
| DZIP1L |  |  |
| FAIM |  |  |
| RNF207 |  |  |
| KCNB1 |  |  |
| NRG2 |  |  |
| GDPD5 |  |  |
| CDA |  |  |
| DMTN |  |  |
| EPB41 |  |  |
| MIS18A |  |  |
| C21orf59 |  |  |
| CSRP1 |  |  |
| RUNX1 |  |  |
| CHAF1B |  |  |
| BTG2 |  |  |
| ATP6V0D1 |  |  |
| LYPD5 |  |  |
| TNFRSF13C |  |  |
| ZBTB8A |  |  |
| ZNF362 |  |  |
| HSF2BP |  |  |
| G6PD |  |  |
| PCNT |  |  |
| ZNF714 |  |  |
| PKN3 |  |  |
| COX6B2 |  |  |
| SHC1 |  |  |
| NLRX1 |  |  |
| TONSL |  |  |
| DMKN |  |  |
| CYGB |  |  |
| ALDH16A1 |  |  |
| ITGA5 |  |  |
| PLCD3 |  |  |
| CXCL16 |  |  |
| WDR90 |  |  |
| TEDC2 |  |  |
| TAF6L |  |  |
| LRP5 |  |  |
| TPCN2 |  |  |
| CMPK1 |  |  |
| C1orf123 |  |  |
| USP24 |  |  |
| DHRS3 |  |  |
| TSSK3 |  |  |
| TMCO4 |  |  |
| MEGF6 |  |  |
| USP1 |  |  |
| ATXN7L2 |  |  |
| ATF3 |  |  |
| IER5 |  |  |
| IL24 |  |  |
| FCMR |  |  |
| PQLC3 |  |  |
| SLC16A14 |  |  |
| CDC42EP3 |  |  |
| LCE3D |  |  |
| TGFA |  |  |
| ALPP |  |  |
| ANTXR2 |  |  |
| GPR155 |  |  |
| KBTBD8 |  |  |
| SLC22A15 |  |  |
| IGFN1 |  |  |
| ELF3 |  |  |
| IGFBP7 |  |  |
| CXCR1 |  |  |
| TGFBR2 |  |  |
| PPM1L |  |  |
| CD200R1 |  |  |
| CCDC191 |  |  |
| TIPARP |  |  |
| IL17RC |  |  |
| PCOLCE2 |  |  |
| UCN |  |  |
| PLB1 |  |  |
| KIF15 |  |  |
| CDCP1 |  |  |
| DTX3L |  |  |
| LIPH |  |  |
| ERMAP |  |  |
| PLXNB1 |  |  |
| HSPA4L |  |  |
| DUSP7 |  |  |
| HMGB2 |  |  |
| ANXA5 |  |  |
| ITGA2 |  |  |
| F2RL2 |  |  |
| CMBL |  |  |
| F2RL1 |  |  |
| ERAP2 |  |  |
| TERT |  |  |
| SHROOM1 |  |  |
| GJB7 |  |  |
| CITED2 |  |  |
| IL31RA |  |  |
| STK17A |  |  |
| SAP30L |  |  |
| KCNK5 |  |  |
| STEAP1 |  |  |
| SYTL3 |  |  |
| IQUB |  |  |
| HEY1 |  |  |
| FABP5 |  |  |
| SUN3 |  |  |
| TMEM74 |  |  |
| TMEM184A |  |  |
| GEM |  |  |
| RPP25L |  |  |
| SYK |  |  |
| ABCA1 |  |  |
| LETM2 |  |  |
| TRPV6 |  |  |
| ANKS6 |  |  |
| METTL27 |  |  |
| C9orf84 |  |  |
| AQP3 |  |  |
| OTUD1 |  |  |
| ZCCHC24 |  |  |
| PGM2L1 |  |  |
| PHYHIPL |  |  |
| C10orf10 |  |  |
| TMEM63C |  |  |
| NSD1 |  |  |
| STOX1 |  |  |
| RNASE7 |  |  |
| E2F7 |  |  |
| LARGE2 |  |  |
| TC2N |  |  |
| HACD1 |  |  |
| TCP11L2 |  |  |
| SPRED1 |  |  |
| SPINT1 |  |  |
| CKB |  |  |
| PCBD1 |  |  |
| STXBP4 |  |  |
| PLEKHF1 |  |  |
| C11orf74 |  |  |
| PPFIBP2 |  |  |
| SERPINB7 |  |  |
| SERPINB8 |  |  |
| ST5 |  |  |
| CENPN |  |  |
| ATF7IP2 |  |  |
| MMP10 |  |  |
| PLK1 |  |  |
| STX3 |  |  |
| PIP4K2C |  |  |
| DIS3L |  |  |
| RCCD1 |  |  |
| MAPRE2 |  |  |
| TEF |  |  |
| CDK12 |  |  |
| ZNF23 |  |  |
| RHEBL1 |  |  |
| TUBA1A |  |  |
| NFKBID |  |  |
| TMC4 |  |  |
| TMEM145 |  |  |
| DNAAF3 |  |  |
| DAPK3 |  |  |
| NXN |  |  |
| SLC43A2 |  |  |
| SERPINF2 |  |  |
| KLK6 |  |  |
| IGFBP6 |  |  |
| MRPL58 |  |  |
| EVPL |  |  |
| MLST8 |  |  |
| ECI1 |  |  |
| ABCA3 |  |  |
| SRRM2 |  |  |
| VWCE |  |  |
| RAB3IL1 |  |  |
| TTC21A |  |  |
| SCARA3 |  |  |
| PBK |  |  |
| NUDT16L1 |  |  |
| IRF2BP2 |  |  |
| HIST1H1E |  |  |
| DTYMK |  |  |
| ING5 |  |  |
| MLKL |  |  |
| HR |  |  |
| CCDC110 |  |  |
| SERINC2 |  |  |
| TMEM223 |  |  |
| IL7R |  |  |
| SEMA4C |  |  |
| CNNM3 |  |  |
| TSPAN5 |  |  |
| STXBP6 |  |  |
| HNRNPH1 |  |  |
| ACTBL2 |  |  |
| DHRSX |  |  |
| C8orf46 |  |  |
| ASMTL |  |  |
| SH3TC2 |  |  |
| ADRB2 |  |  |
| B3GALNT1 |  |  |
| PTAFR |  |  |
| CXCL8 |  |  |
| RASSF6 |  |  |
| SPRR1B |  |  |
| CLIC4 |  |  |
| HEXDC |  |  |
| AGPAT2 |  |  |
| ASPSCR1 |  |  |
| FASN |  |  |
| MT1E |  |  |
| DCXR |  |  |
| CSGALNACT2 |  |  |
| AVEN |  |  |
| TRIM56 |  |  |
| REPS2 |  |  |
| KLF13 |  |  |
| IFFO2 |  |  |
| CHD3 |  |  |
| ALCAM |  |  |
| ADRA1B |  |  |
| CMTM8 |  |  |
| FABP4 |  |  |
| B3GNT2 |  |  |
| TCAF2 |  |  |
| GPRC5C |  |  |
| PYM1 |  |  |
| TMC7 |  |  |
| CAVIN4 |  |  |
| PLAC1 |  |  |
| C11orf24 |  |  |
| INSR |  |  |
| TMEM37 |  |  |
| LRG1 |  |  |
| CANT1 |  |  |
| CHST11 |  |  |
| KRT15 |  |  |
| KRT13 |  |  |
| KSR2 |  |  |
| DSEL |  |  |
| ASXL1 |  |  |
| DLK2 |  |  |
| LRRC8C |  |  |
| PTGER4 |  |  |
| BCL2L1 |  |  |
| CXXC5 |  |  |
| PIK3CD |  |  |
| ENC1 |  |  |
| BPTF |  |  |
| RGS19 |  |  |
| GATM |  |  |
| RHNO1 |  |  |
| ZNF540 |  |  |
| MLLT3 |  |  |
| C20orf196 |  |  |
| SYNPO |  |  |
| RAB33B |  |  |
| QARS |  |  |
| KLF11 |  |  |
| EIF2AK3 |  |  |
| CALB2 |  |  |
| CERS6 |  |  |
| CFAP53 |  |  |
| C2CD2L |  |  |
| GNG12 |  |  |
| FGGY |  |  |
| C2orf54 |  |  |
| ZMAT3 |  |  |
| LRRC20 |  |  |
| RARG |  |  |
| MYEOV |  |  |
| OXSR1 |  |  |
| PARP14 |  |  |
| AHSA2 |  |  |
| ABLIM3 |  |  |
| SNCG |  |  |
| TRIB1 |  |  |
| SSSCA1 |  |  |
| PTPRM |  |  |
| NABP1 |  |  |
| CCDC106 |  |  |
| SLC19A1 |  |  |
| HEG1 |  |  |
| PLK3 |  |  |
| SPTBN2 |  |  |
| CEP19 |  |  |
| MSRB3 |  |  |
| TLR6 |  |  |
| CTU2 |  |  |
| ADCY6 |  |  |
| MRPL11 |  |  |
| ANGEL2 |  |  |
| B4GAT1 |  |  |
| SH3PXD2B |  |  |
| FAM241A |  |  |
| FZD4 |  |  |
| CNIH2 |  |  |
| UBE2C |  |  |
| PDIK1L |  |  |
| MARCKSL1 |  |  |
| YPEL2 |  |  |
| CSRP2 |  |  |
| MED16 |  |  |
| CST6 |  |  |
| ZNF25 |  |  |
| CLCF1 |  |  |
| LIPT2 |  |  |
| FOSL1 |  |  |
| ETV4 |  |  |
| TMEM270 |  |  |
| DENND2C |  |  |
| B3GALT6 |  |  |
| DMRTA1 |  |  |
| BDNF |  |  |
| CDK5R1 |  |  |
| NCKAP5 |  |  |
| TYMS |  |  |
| SHMT1 |  |  |
| DPP7 |  |  |
| DEAF1 |  |  |
| ACER2 |  |  |
| POLE |  |  |
| FAM210A |  |  |
| ACOT4 |  |  |
| ZBED2 |  |  |
| ATOX1 |  |  |
| PIDD1 |  |  |
| JUN |  |  |
| GBA |  |  |
| CD163L1 |  |  |
| TMEM94 |  |  |
| AP3S1 |  |  |
| STAP2 |  |  |
| ZNF114 |  |  |
| PARD6G |  |  |
| KDELC2 |  |  |
| WDR6 |  |  |
| BEND3 |  |  |
| TUBAL3 |  |  |
| P4HTM |  |  |
| CTNNBIP1 |  |  |
| GTPBP6 |  |  |
| ZNF713 |  |  |
| PARP10 |  |  |
| KCTD12 |  |  |
| APOLD1 |  |  |
| C17orf62 |  |  |
| AURKB |  |  |
| KLHDC7A |  |  |
| TRIML2 |  |  |
| TMTC2 |  |  |
| HES7 |  |  |
| FUCA1 |  |  |
| PLD6 |  |  |
| LACC1 |  |  |
| ARL14 |  |  |
| APOBEC3B |  |  |
| CITED4 |  |  |
| ABCA13 |  |  |
| TSHZ1 |  |  |
| CCDC43 |  |  |
| FZD2 |  |  |
| MTURN |  |  |
| MCFD2 |  |  |
| HIST1H2AC |  |  |
| HIST1H2BC |  |  |
| HOXC9 |  |  |
| D2HGDH |  |  |
| GPR137C |  |  |
| BBS12 |  |  |
| RPH3AL |  |  |
| F2R |  |  |
| MUC16 |  |  |
| PJA1 |  |  |
| POLR2A |  |  |
| TMEM45A |  |  |
| RAP2B |  |  |
| TNFSF15 |  |  |
| PLAG1 |  |  |
| C5orf30 |  |  |
| AMIGO1 |  |  |
| RELL1 |  |  |
| IBA57 |  |  |
| ERCC6L2 |  |  |
| ATP6AP2 |  |  |
| UBE2E2 |  |  |
| SYNM |  |  |
| NLRP10 |  |  |
| AP1S2 |  |  |
| DCAF4L1 |  |  |
| C1S |  |  |
| KBTBD3 |  |  |
| PLCXD1 |  |  |
| ADI1 |  |  |
| CSF1R |  |  |
| ANXA2 |  |  |
| HCAR2 |  |  |
| ALG12 |  |  |
| ZNF721 |  |  |
| PYCR1 |  |  |
| SLC25A10 |  |  |
| CAMK1D |  |  |
| GAS6 |  |  |
| EPHA10 |  |  |
| CMTM4 |  |  |
| ACTG1 |  |  |
| FAM120C |  |  |
| NR2C2AP |  |  |
| CRELD2 |  |  |
| HIST2H2AC |  |  |
| HIST2H2AB |  |  |
| HIST1H1B |  |  |
| PKP3 |  |  |
| CSF1 |  |  |
| TOP1MT |  |  |
| PTP4A3 |  |  |
| PROS1 |  |  |
| DUSP8 |  |  |
| TMEM173 |  |  |
| FAM19A3 |  |  |
| NELL2 |  |  |
| MED12 |  |  |
| AMER1 |  |  |
| FAM110C |  |  |
| OSBP2 |  |  |
| PRR16 |  |  |
| BTBD6 |  |  |
| RBM43 |  |  |
| SUMO3 |  |  |
| JAG2 |  |  |
| FMNL1 |  |  |
| FAM227A |  |  |
| USP18 |  |  |
| TMEM106A |  |  |
| CA13 |  |  |
| MAFF |  |  |
| FLRT2 |  |  |
| ANO9 |  |  |
| HIST1H2BL |  |  |
| IFITM2 |  |  |
| ZNF74 |  |  |
| ARL15 |  |  |
| METTL7A |  |  |
| TMEM179B |  |  |
| ROR1 |  |  |
| MUC1 |  |  |
| AHNAK2 |  |  |
| OLFML2A |  |  |
| ZFP36L1 |  |  |
| PMEL |  |  |
| MYBL1 |  |  |
| PIGP |  |  |
| NAT8L |  |  |
| GNB1L |  |  |
| LAMP1 |  |  |
| IRS2 |  |  |
| BICD2 |  |  |
| TMLHE |  |  |
| ZNF284 |  |  |
| ANKRD46 |  |  |
| KIF18B |  |  |
| SAPCD2 |  |  |
| EDARADD |  |  |
| ZNF75D |  |  |
| GLDN |  |  |
| KPNA4 |  |  |
| INSIG1 |  |  |
| SMIM29 |  |  |
| PDE2A |  |  |
| CYP27C1 |  |  |
| KRT16 |  |  |
| HEXIM1 |  |  |
| KRT14 |  |  |
| PDZD7 |  |  |
| MITF |  |  |
| MT1X |  |  |
| EPOR |  |  |
| ISG15 |  |  |
| SAMD11 |  |  |
| SPRY4 |  |  |
| THSD4 |  |  |
| SEMA4D |  |  |
| HIST1H1C |  |  |
| FAM122A |  |  |
| ARHGAP11B |  |  |
| DNER |  |  |
| ZSCAN23 |  |  |
| TPRG1 |  |  |
| ARL4C |  |  |
| C6orf132 |  |  |
| MAPK12 |  |  |
| ZC3H6 |  |  |
| PRKAR1B |  |  |
| NCR3LG1 |  |  |
| GTF2F2 |  |  |
| ZP3 |  |  |
| C15orf52 |  |  |
| FAM72B |  |  |
| NANOS1 |  |  |
| DPYD |  |  |
| TMEM120B |  |  |
| SPRED3 |  |  |
| ENTPD8 |  |  |
| ZNF33A |  |  |
| MAOA |  |  |
| PNRC2 |  |  |
| S100A14 |  |  |
| SH2D5 |  |  |
| IL1RAP |  |  |
| AKR1C3 |  |  |
| FAT4 |  |  |
| SEMA4A |  |  |
| SIRPB2 |  |  |
| ZBTB44 |  |  |
| STK31 |  |  |
| TRRAP |  |  |
| PTPN1 |  |  |
| PPP1R26 |  |  |
| TPK1 |  |  |
| SLC6A9 |  |  |
| MAN2A2 |  |  |
| HRH1 |  |  |
| ZNF33B |  |  |
| GM2A |  |  |
| HIST1H2AI |  |  |
| S100A2 |  |  |
| CD47 |  |  |
| MAML3 |  |  |
| HIST1H2AG |  |  |
| MVB12B |  |  |
| ARID5A |  |  |
| KIAA1211L |  |  |
| SCN8A |  |  |
| LAMB3 |  |  |
| FAM3C |  |  |
| LAGE3 |  |  |
| WDR45 |  |  |
| HIST1H4C |  |  |
| IGF2R |  |  |
| PCBP2 |  |  |
| SRC |  |  |
| ACSL5 |  |  |
| ZNF785 |  |  |
| PIWIL2 |  |  |
| ZNF165 |  |  |
| ZNF720 |  |  |
| DACT3 |  |  |
| IPP |  |  |
| STMN3 |  |  |
| C5orf56 |  |  |
| TTC30A |  |  |
| COL4A6 |  |  |
| C5orf42 |  |  |
| SERPINB2 |  |  |
| SERPINB13 |  |  |
| PHF2 |  |  |
| PSAP |  |  |
| HOXC6 |  |  |
| GPAA1 |  |  |
| SGTB |  |  |
| HIST1H2BK |  |  |
| ERO1A |  |  |
| S100A6 |  |  |
| SIRPA |  |  |
| AKR1B10 |  |  |
| HMGN5 |  |  |
| DDRGK1 |  |  |
| ZXDA |  |  |
| SLC29A3 |  |  |
| HYLS1 |  |  |
| ZNF442 |  |  |
| HOXC4 |  |  |
| PIM3 |  |  |
| SPRED2 |  |  |
| NRARP |  |  |
| TPM2 |  |  |
| SH3BGRL2 |  |  |
| ATL1 |  |  |
| MDM4 |  |  |
| TUSC1 |  |  |
| DLL1 |  |  |
| PPP1R14C |  |  |
| F5 |  |  |
| FAM169A |  |  |
| ARHGAP11A |  |  |
| HMGN2 |  |  |
| GRK5 |  |  |
| TBKBP1 |  |  |
| L3MBTL3 |  |  |
| TGM2 |  |  |
| CHML |  |  |
| FAM229B |  |  |
| HIST2H2BF |  |  |
| RBM20 |  |  |
| SOX18 |  |  |
| LIME1 |  |  |
| RTL8A |  |  |
| PSMB8 |  |  |
| DXO |  |  |
| ZBTB12 |  |  |
| NEU1 |  |  |
| FAM216A |  |  |
| FBXO48 |  |  |
| PSG5 |  |  |
| ZNF783 |  |  |
| SPIRE2 |  |  |
| ATP6AP1L |  |  |
| TMSB4X |  |  |
| ITPRIPL2 |  |  |
| KLRC3 |  |  |
| SERPINB5 |  |  |
| CFAP44 |  |  |
| KRTAP2-3 |  |  |
| CRYGS |  |  |
| QTRT1 |  |  |
| ARHGAP19 |  |  |
| NDUFS3 |  |  |
| KCTD11 |  |  |
| CSNK1E |  |  |
| ITGA1 |  |  |
| AP1G2 |  |  |
| REPIN1 |  |  |
| PRCD |  |  |
| ZBED1 |  |  |
| HOMEZ |  |  |
| NPEPL1 |  |  |
| FXYD7 |  |  |
| APOL6 |  |  |
| FADS3 |  |  |
| BTBD19 |  |  |
| RFPL4A |  |  |
| SMIM13 |  |  |
| CT62 |  |  |
| HSBP1L1 |  |  |
| KLLN |  |  |
| RFPL4AL1 |  |  |
| ANKRD18B |  |  |
| PSG1 |  |  |
| ZSCAN31 |  |  |
| FAM200B |  |  |
| TXNDC5 |  |  |
| C1orf226 |  |  |
| LCE1F |  |  |
| TMEM189 |  |  |
| PLCXD2 |  |  |
| C22orf39 |  |  |
| ARPIN |  |  |
| CFAP57 |  |  |
| ETV5 |  |  |
| CEBPA |  |  |
| ZCCHC3 |  |  |
| TWF2 |  |  |
| FMN1 |  |  |
| TMEM158 |  |  |
| FOXD1 |  |  |
| HOXA10 |  |  |
| TRNP1 |  |  |
| MEX3A |  |  |
| DPP3 |  |  |
| EID1 |  |  |
| CHURC1 |  |  |
| INAFM2 |  |  |
| MMP12 |  |  |
| GTF2I |  |  |
| MSMB |  |  |
| BAHCC1 |  |  |
| C17orf113 |  |  |
| FBXO17 |  |  |
| SLC25A53 |  |  |
| SRXN1 |  |  |
| LIX1L |  |  |
| ANKRD34A |  |  |
| POM121C |  |  |
| HIST1H2BM |  |  |
| HIST1H3G |  |  |
| NATD1 |  |  |
| HIST1H3B |  |  |
| HIST1H2BO |  |  |
| HIST1H3E |  |  |
| HIST1H2AH |  |  |
| HIST1H2BH |  |  |
| UHRF1 |  |  |
| RAB7B |  |  |
| PIGW |  |  |
| HIST1H2BF |  |  |
| HIST1H3F |  |  |
| MYO19 |  |  |
| HIST1H2AB |  |  |
| DHRS11 |  |  |
| HIST1H4A |  |  |
| HIST1H4B |  |  |
| RP11-437B10.1 |  |  |
| C2orf81 |  |  |
