# Supplementary material for: Pharmacological targeting of TNS3 with histone deacetylase inhibitor as a therapeutic strategy in esophageal squamous cell carcinoma
Source: Aging (Albany NY). 2021 May 28;13(11):15336–52. doi: 10.18632/aging.203091 (PMC8221360; doi:10.18632/aging.203091)
Supplement: Supplementary Tables 2, 3 and 4 [file aging-13-203091-s003.pdf]

SUPPLEMENTARY TABLES

Supplementary Table 2. Sequences of primers used in qRT-PCR.

Gene	Forward primer	Reverse primer
TNS3	ACCTCACTTACATCACGGAGC	GTAGGTTGTGCAGGTAGGACT
ABLIM3	ACATCAGTGGCAGAGTCTTGG	TCCTTCGGTGAACATCTGGTG
EMP1	ATTCTTGTGGGGGTGTCCATC	CAGGACCAGATAGAGAACGCC
OXSR1	AGCCATGAGTCAATGCCATCA	ACAGAACCTCCACTTAGCAGC
MARCKSL1	GCTAGTGCAGCCTCAGAAGAA	CTACTCATTCTGCTCAGCGCT
KIF20A	CCCACACGATTCAAGGTACCA	GGCTTCAGATCAGGTGTTGGA
NCAPG2	CTGTGGACAAGAGCTACTGCA	CAGCCAGTTGTCAACAAGCTC
PUS7	AGCCTACAGGGAAATGCTCAC	ATGCAACGACTTCCCAGCTAA
HMMR	TCTTCTACAGGAACGTGGTGC	GTGCAGCATTTAGCCTTGCTT
EIF2AK2	AGCACACTCGCTTCTGAATCA	AGGTCAAATCTGGGTGCCAAA
GAPDH	GGAGCGAGATCCCTCCAAAAT	GGCTGTTGTCATACTTCTCATGG

Supplementary Table 3. Sequences of siRNA used in siRNA library and transfection.

Gene	siRNA #1	siRNA #2	siRNA #3
TNS3	GTACAAGGCGGATATTTCA	GGTTGTAGCTCACCAGTAT	GGATCTGCATCGTCATCGA
ABLIM3	TCAGGCTTCTTCTTCAAGA	GGAATGAACTGAAGAAGCA	CCACTTCCACATCAGATGC
EMP1	GCTTCATCATCGGCGTTCT	GCAGTGACAGCCTGTCATA	CCACATCGCTACTGTTATT
OXSR1	GCAGCAATTTCACAACTCA	GCACCAACCATTTCTGAAA	GGATCAGGTTCACAAGAAA
MARCKSL1	CCTTCAAGAGAAATCGGAA	TCAAGAAGCCTTTCAAATT	TCTCTTTCAAGAAGCCTTT
KIF20A	GCATCCTTCTTCAACCTAA	CCATCTGGATCTCATTCTT	GGATCTCATTCTTTGAGAT
NCAPG2	GCCTGCACCAACATAGCAA	CCTTTCAGCCTAAATGAAT	GCTTCCAGAGTATCTGAAA
PUS7	GCACTGGTTGTCGAAGATA	CCATCATCCATCAGGCTAT	GCTAGGGAATTTCAGCTAT
HMMR	CAGCTGGAAGATGAAGAAG	TGGAAGATGAAGAAGGAAG	GGAAGATGAAGAAGGAAGA
EIF2AK2	CCTGAGACCAGTGATGATT	GGACCTTGGAACAATGGAT	GCTAATTCTTGCTGAACTT
NC	TTCTCCGAACGTGTCACGT		

Notes: All target sequence are listed as 5′-3′.

Supplementary Table 4. Sequences of shRNA used in plasmid construction.

Vector	Gene	AgeI (5′-3′)	EcoRI (5′-3′)
pLKO.1-EGFP-puro	sh*TNS3*_1	CCGGGTACAAGGCGGATATTTCACTC<br>GAGTGAAATATCCGCCTTGTACTTTTT	AATTAAAAAGTACAAGGCGGATATTTC<br>ACTCGAGTGAAATATCCGCCTTGTAC
	sh*TNS3*_2	CCGGGGTTGTAGCTCACCAGTATCTCG<br>AGATACTGGTGAGCTACAACCTTTTT	AATTAAAAAGGTTGTAGCTCACCAGTA<br>TCTCGAGATACTGGTGAGCTACAACC
	shNC	CCGGTTCTCCGAACGTGTCACGTCTCG<br>AGACGTGACACGTTCGGAGAATTTTT	AATTAAAAATTCTCCGAACGTGTCACG<br>TCTCGAGACGTGACACGTTCGGAGAA
